# Simulating future supply of and requirements for human resources for health in high-income OECD countries

**DOI:** 10.1186/s12960-016-0168-x

**Published:** 2016-12-12

**Authors:** Gail Tomblin Murphy, Stephen Birch, Adrian MacKenzie, Janet Rigby

**Affiliations:** 1 Dalhousie University, 5869 University Avenue, Halifax, Nova Scotia B3H 4R2 Canada; 2 McMaster University, 1280 Main Street West, Hamilton, Ontario L8S 4K1 Canada

**Keywords:** HRH planning, Health workforce planning, Health workforce requirements, OECD countries, High-income countries, Midwives, Nurses, Physicians

## Abstract

**Background:**

As part of efforts to inform the development of a global human resources for health (HRH) strategy, a comprehensive methodology for estimating HRH supply and requirements was described in a companion paper. The purpose of this paper is to demonstrate the application of that methodology, using data publicly available online, to simulate the supply of and requirements for midwives, nurses, and physicians in the 32 high-income member countries of the Organisation for Economic Co-operation and Development (OECD) up to 2030.

**Methods:**

A model combining a stock-and-flow approach to simulate the future supply of each profession in each country—adjusted according to levels of HRH participation and activity—and a needs-based approach to simulate future HRH requirements was used. Most of the data to populate the model were obtained from the OECD’s online indicator database. Other data were obtained from targeted internet searches and documents gathered as part of the companion paper.

**Results:**

Relevant recent measures for each model parameter were found for at least one of the included countries. In total, 35% of the desired current data elements were found; assumed values were used for the other current data elements. Multiple scenarios were used to demonstrate the sensitivity of the simulations to different assumed future values of model parameters. Depending on the assumed future values of each model parameter, the simulated HRH gaps across the included countries could range from shortfalls of 74 000 midwives, 3.2 million nurses, and 1.2 million physicians to surpluses of 67 000 midwives, 2.9 million nurses, and 1.0 million physicians by 2030.

**Conclusions:**

Despite important gaps in the data publicly available online and the short time available to implement it, this paper demonstrates the basic feasibility of a more comprehensive, population needs-based approach to estimating HRH supply and requirements than most of those currently being used. HRH planners in individual countries, working with their respective stakeholder groups, would have more direct access to data on the relevant planning parameters and would thus be in an even better position to implement such an approach.

**Electronic supplementary material:**

The online version of this article (doi:10.1186/s12960-016-0168-x) contains supplementary material, which is available to authorized users.

## Background

Human resources for health (HRH) are a core element of health systems. Their availability, accessibility, quality, and performance directly impact the effectiveness and equity of health care services [1]. Planning for HRH therefore has a central role in health care systems. According to one of the early seminal texts on the subject, HRH planning is,

…the process of estimating the number of persons and the kind of knowledge, skills, and attitudes they need to achieve predetermined health targets and ultimately health status objectives. Such planning also involves specifying who is going to do what, when, where, how, and with what resources for what population groups or individuals so that the knowledge and skills necessary for the adequate performance can be made available according to predetermined policies and time schedules. This planning must be a continuing and not a sporadic process, and it requires continuous monitoring and evaluation [2].

Effective HRH planning entails matching the HRH supply with the requirements for HRH necessary to satisfy health care system objectives. In many publicly funded health care systems, these objectives relate to meeting the health care needs of the population and involve replacing traditional measures of demand for HRH (determined by a population’s ability and willingness to pay for the services HRH provide) with measures of the HRH required to support service planning and delivery in ways that address a population’s need for care. Under such an approach, the quantity and type of services planned to respond to those needs must be determined in the context of a government’s capacity to fund care. It may be that not all needs for care can be met or that care levels are less than “gold-standard” or evidence-based levels because of resource limitations, but health care services, and the HRH required to deliver them, are still planned in relation to the levels and distribution of needs for care in the population. This is distinct from more common approaches where estimates of HRH requirements are based simply on service levels observed by demographic characteristics in the population.

HRH planning remains a major challenge in many countries [3–6] despite its centrality to the success of global campaigns such as the Millennium Development Goals [1]. Recent efforts by the World Health Organization (WHO), the Global Health Workforce Alliance (GHWA), and partner organizations to facilitate the development of a global HRH strategy for the period 2016–2030 [7] reflect the growing recognition of the importance of HRH planning. To inform the development of this strategy, several research teams were commissioned to provide multiple, complementary pieces of evidence pertaining to the current and future HRH situations in countries around the world. The WHO/Pan American Health Organization (PAHO) Collaborating Centre on Health Workforce Planning and Research at Dalhousie University was commissioned by the WHO and the GHWA to produce two papers on the HRH situations in member countries of the Organisation for Economic Co-operation and Development (OECD) who are classified as “high income” by the World Bank. These countries include Australia, Austria, Belgium, Canada, Chile, the Czech Republic, Denmark, Estonia, Finland, France, Germany, Greece, Hungary, Iceland, Ireland, Israel, Italy, Japan, Luxembourg, the Netherlands, New Zealand, Norway, Poland, Portugal, Slovakia, Slovenia, South Korea, Spain, Sweden, Switzerland, the United Kingdom, and the United States (referred to hereafter as the “included countries”)^1^. Other research teams, using different methods, produced other evidence (e.g. pertaining to low- and middle-income countries).

The first paper [8] described a rapid review and synthesis of recent analyses of HRH requirements and labor market dynamics in the included countries. Although over 200 relevant documents were reviewed in detail as part of this first phase, collectively, they do not include sufficient information to provide a clear picture of the expected future HRH situation in these countries. According to most of the analyses reviewed, HRH supply in these countries is generally expected to grow, but different analyses reach different conclusions about future HRH country requirements. It is not clear whether that expected growth in supply will be adequate to meet health system objectives in the future—that is, whether there will be surpluses or shortages of various professions in various countries. Although most of the reviewed analyses suggest that the numbers of physicians and nurses required in the included countries are likely to increase more rapidly than supply in the future, resulting in shortages, this view varies across analyses depending on the methods and assumptions used. For example, one analysis [9] projected a shortage of over 900 000 registered nurses (RNs) in the United States by 2030, while another [10], using different methods and assumptions, estimated a surplus of over 300 000 RNs in the USA by 2025. Further, most analyses for professions other than nurses and physicians suggest that their respective supplies are likely to be greater than required in the future. The implications of these respective expected surpluses and shortages across professions, in terms of meeting health system objectives, are not clear from the evidence reviewed.

More broadly, the review results suggest that HRH policy questions in these countries tend to be developed from existing data and analytical methods as opposed to new data and analytical methods being developed to address HRH policy questions. In an attempt to inform estimates of future HRH needs, seven criteria for identifying an HRH planning approach appropriate to a given country or jurisdiction were identified in the first report:

The approach is consistent with the objectives of the health system. This means, for example, that a system whose objective involves addressing the health care needs of its population must use an HRH planning method that estimates HRH requirements as a function of population health measures so that resources can be planned in accordance with levels of—and potential changes in—the population’s needs for health care. Resources are then allocated between populations based on differences in needs between those populations and increased or decreased over time in accordance with increases or decreases in those needs, while also allowing for changes in the way needs are to be met (e.g., using new technologies or different types of health care teams). Although meeting population health care needs is a goal shared by many health care systems, the findings of the review indicated that few countries appear to be using needs-based methods for HRH planning. Instead, the HRH analyses reviewed appear to be using utilization- or supply-based approaches.
(a) HRH requirements are derived from service requirements; and
  (b) Those service requirements are aligned with system objectives (e.g., addressing population needs for care arising from various diseases or other health issues). Requirements for HRH are a manifestation of requirements for the services they provide. Hence, estimates of HRH requirements must be derived from estimates of the requirements for those services. This makes it possible to consider and plan for potential future changes in the way services are delivered resulting from new technologies, changes in scopes of practice, and so on. The results of this review show, however, that HRH planning approaches that cannot account for such changes—such as the use of provider/population ratios—remain prevalent.
The approach considers HRH requirements in the context of production functions for health services (i.e., dependent upon the availability or use of facilities and other non-human inputs to service production and on models of care to be used). Although the availability of HRH is important to the delivery of health care services, other types of human and non-human resources - such as facilities, equipment, and medications - are also necessary. Effective health system planning approaches must recognize this dependency by considering how the availability (or lack thereof) of (a) other HRH and (b) non-human resources may affect their collective production of health care services, including the potential for substitution of one type of resource for another. For example, the availability of operating theater nurses or operating theaters may impact on the volume of surgeries that surgeons can perform, even if the number of surgeons and the hours they work remain the same. The review conducted in the first paper found several examples of approaches that explicitly incorporated this potential for different types of HRH. However, although documents sometimes acknowledged the influence of the availability of non-human resources on HRH requirements, no analyses that directly incorporated this relationship were found.
The approach explicitly considers the role and determinants of productivity (i.e., units of service per hour of work). In order to translate health care service requirements into HRH requirements, HRH planners must consider the rate at which different types of HRH are able to provide those services per unit time—i.e., their productivity—under a given set of circumstances. Numerous analyses found by the review explicitly included productivity as part of their calculations. Although the contexts in which productivity was considered varied widely across these documents, they generally showed that projections regarding the future HRH situation are highly sensitive to even small differences in levels of HRH productivity.
HRH supply is measured in terms of time devoted to service delivery (i.e., flow generated by a stock of HRH) as opposed to focussing only on the HRH stock (numbers of HRH). The availability of health care services is determined by a number of factors (e.g., participation in direct care provision as opposed to administration) in addition to the raw “stock” or head count of different types of HRH available to provide them. The review found analyses from many countries demonstrating how changes or differences in these factors can have profound effects on the effective supply of HRH, and most of the HRH supply analyses found through the review considered at least one of these, most frequently hours worked. Several analyses, however, did not take any of these factors into account and instead estimated HRH supply based solely on head counts.
The approach considers the determinants of flow (e.g., hours worked) and stock (entries/exits) as policy variables. The factors that determine the stock and flow of HRH supply, such as the amount of time spent providing patient care (activity levels) and the proportion of licensed HRH who are actively practicing (participation levels), are sensitive—to varying degrees—to HRH policies such as education and payment models. Most of the analyses of HRH supply found through the review reflected this situation; in some cases, such factors were the primary focus of the analyses.
The approach considers (a) the cost implications of HRH plans and (b) the extent to which HRH plans are aligned with health system financial planning. Essential to determining the relative appropriateness of any potential HRH policy is an understanding of its financial implications in the broader context of the jurisdictional fiscal situation. Although many of the documents included in the review acknowledged this point, comparatively few explicitly incorporated financial considerations into their analyses.

Although none of the approaches examined in the review met all of these criteria, several approaches applied in different contexts within three countries—Australia, Canada, and New Zealand—met all but one. Further, because the individual criteria specified may have different levels of importance in different planning contexts, additional examples of other approaches which met each individual criterion were also identified so that planners can explore different options depending on their respective planning priorities. The development of this data-driven approach to simulating future HRH supply and requirements is intended to inform the early identification of trends toward shortfalls or surpluses and to identify policy levers through which adjustments can be made to better match future supply and requirements.

### Objective

The objective of this second paper is to provide simulations of the future supply of and needs-based requirements for midwives, nurses, and physicians in high-income OECD countries up to the year 2030 using a methodology as consistent as possible with the criteria identified in the first paper.

## Methods

Based on these criteria, a simulation of future HRH supply in terms of head counts was produced using a stock-and-flow approach, which entails adjusting current HRH stocks according to expected flows in (e.g., new graduates, inward migration) and out (e.g., retirements, attrition to other sectors, outward migration) of each country’s stock. The results of the review indicated that this is a widely used method of modeling HRH supply (e.g., [9–11]). In line with criteria 5 and 6, these head counts are then adjusted according to levels of participation (providing direct patient care) and activity (proportion of full-time hours spent providing direct patient care) for different types of HRH. For example, a country with 10 000 nurses of whom 80% provide at least some direct patient care and who work an average of 60% full-time hours would be deemed to have an effective supply of 4800 full-time equivalent (FTE) nurses. Several documents reviewed as part of the first paper used such adjustments in estimating HRH supply (e.g., [12–14]). Costs of producing and maintaining these stocks can then be simulated based on average training costs and wages (criterion 7); although several of the documents reviewed in the first paper considered HRH remuneration in estimating HRH supply and associated costs (e.g., [15–17]), none included analyses that also considered training costs.

To inform the process of estimating HRH requirements, and in keeping with criterion 1, a review of the objectives of each included country’s health care system, as described in documents obtained for the first report, was conducted. The primary source for this information was the set of health system reviews published by the European Observatory on Health Systems and Policies in their *Health Systems in Transition* series [18], which provide detailed descriptions of the health care systems in most of the included countries. Where possible, this information was supplemented by cross-references with original source documents (such as strategic plans, legislation, or referenced journal articles) or searches on individual national health ministry websites. A table summarizing the objectives of each of the included countries’ health care systems, as described in these documents, is provided as Additional file 1.

**Fig. 1:**
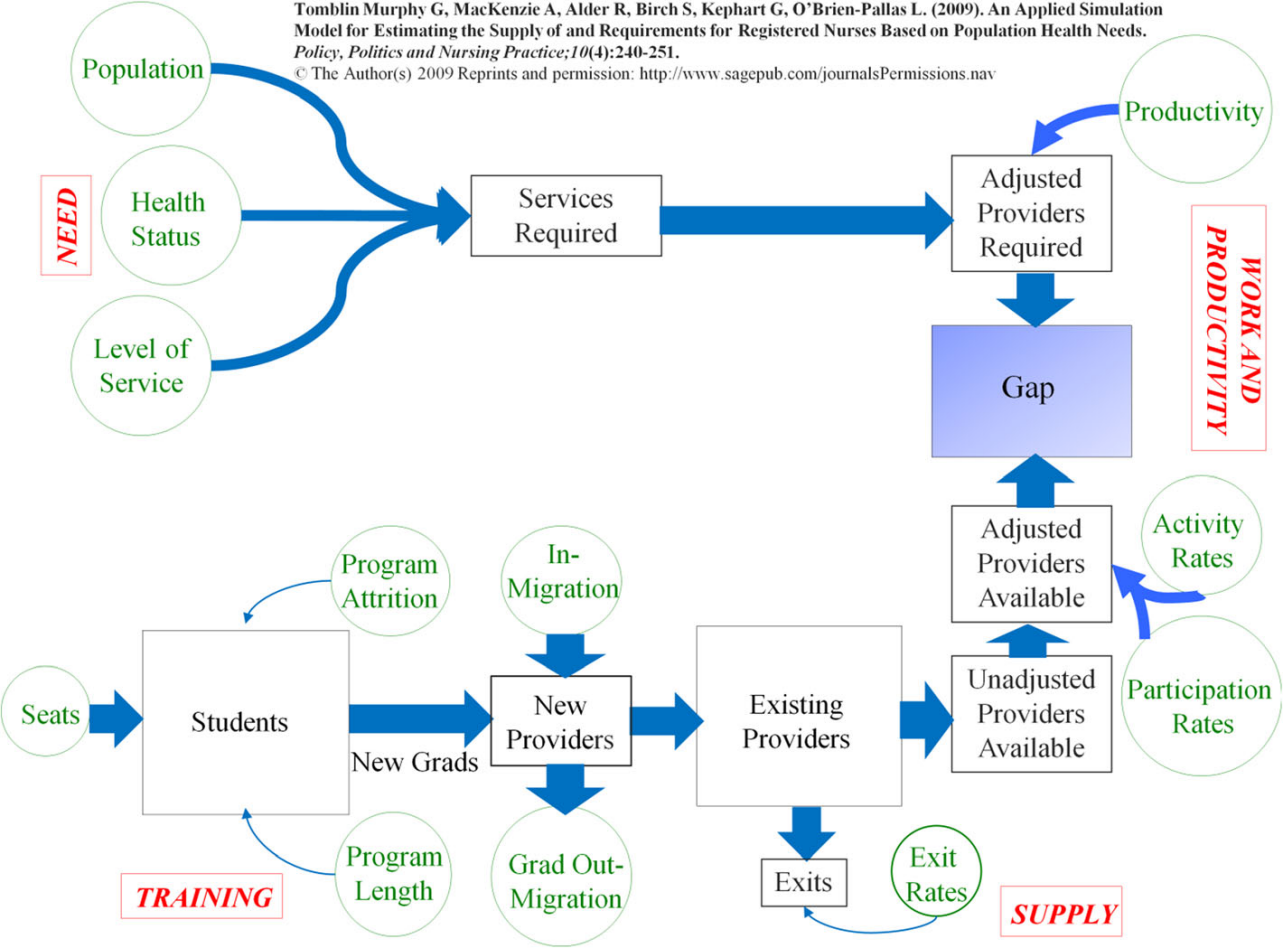
Graphical representation of the approach

Ensuring equitable access to health care services and maintaining and/or promoting the health of their respective populations were the objectives shared across most of the included countries. As such, service requirements were simulated according to different levels of health within countries and existing levels of service provision by those levels of health (i.e., a needs-based approach). This means that this approach differentiates between, for example, the number of physician visits a 75-year-old woman in poor health would require as opposed to a 75-year-old woman with good health. Under more common utilization-based approaches, all 75-year-old women would be assumed to require the same number of physician visits, regardless of their level of health. These service requirements were then converted to simulated HRH requirements (to meet criteria 2) based on estimates of the productivity of different types of HRH (criterion 4). This needs-based approach is consistent with that described in several previous studies (e.g., [19, 20]) where service requirements are estimated by multiplying the size of the population to be served by the distribution of health care needs within that population and the planned number and type of services to be provided per level of health care need. A graphical representation of the approach is provided in Fig. 1. Because ability to pay is only included as a determinant of access to health care by design in one of the included countries (and many others specify that it should not affect access to care), measures of this ability were not included—in other words, a demand-based approach was deemed inappropriate for this work.

In this way, the supply of and requirements for midwives, nurses, and physicians were simulated for each included country from 2015 through 2030. Analyses were conducted at the national level. The bulk of the data used to populate the models for each profession and country was obtained from the OECD’s indicator database [21]. More specifically, data on population projections by age and sex; the distribution of self-assessed health status by age and sex; volumes of hospital days and physician consultations; current supplies of midwives, nurses, and physicians; annual numbers of graduates of each of these professions; annual numbers of in-migrant nurses and physicians; the distribution of physicians by age; and the proportion of licensed members of each profession who are actively practicing were obtained for most of the included countries through the OECD indicator database. Data for these parameters for countries not available through the OECD database, as well as data on other parameters (levels of service provision by level of health status and levels of activity for each profession), were obtained from other existing public online sources such as the WHO’s indicator database [22] or through documents gathered as part of the first paper. In the time available, it was not possible to engage with the various HRH stakeholder groups in the included countries to obtain additional information or to do targeted internet searches for information on each individual country and profession. HRH planners and other relevant stakeholder groups within the included countries would likely be better positioned to obtain this information.

Table 1 provides an overview of the data that were sought—and found—to inform the projections. The values in each cell indicate the number of countries, out of the 32 included in these simulations, for which input data were found for a given parameter, profession, and country. It is not possible to identify the specific sources for each component of each model in each country within the space of the main body of this paper; instead this information is provided in Additional file 2. This analysis was focused only on three types of HRH—midwives, nurses, and physicians—due to data limitations but also because these are the three professions identified by the WHO as being essential for maternal and child health [23]. A more comprehensive HRH analysis would need to also take into account the role of and needs for all other types of HRH.

**Table 1:** Overview of data availability by profession

Planning parameter	Number of countries for which data were found		
Population characteristics			
Population size and projections by age and sex	32		
Health status by age^a^ and sex	31		
Health care system characteristics	Midwife model	Nurse model	Physician model
Health care provision by age, sex, and health status	0	2	2
Service provision per FTE provider per year	32	29	26
Average total annual wages per FTE	0	28	27
Average training cost per graduate	0	0	0
Average replacement cost per FTE	0	1	0
Number of new graduates per year	31	32	32
Age distribution of new graduates	0	1	1
Number of in-migrants per year	3	22	24
Age distribution of in-migrants	0	0	0
Exits per year by age	1	1	1
Total yearly enrolment in training programs	1	1	1
% of students completing training	0	1	1
% of graduates remaining in-jurisdiction	0	1	1
Head count of current supply	31	32	32
Age distribution of current supply	3	3	29
% of licensed workforce providing any patient care	24	19	1
Average hours worked/week by participating workforce	0	1	1

^a^Data on the incidence of low birth weight by mother’s age were not found

In aggregate, 35% of the data elements required to implement the estimation approach were found. This should not be interpreted to mean that the data required to estimate HRH supply and requirements in a manner consistent with the above criteria do not exist. For example, there is information on acute care provision in Canada maintained by the Canadian Institute for Health Information [23] that can be adjusted for acuity and the intensity of care provided but which could not be obtained within the timelines of this project. Planners within individual countries would be more familiar with potential local sources of the information required to provide a stronger base for the simulations.

Although online resources such as the OECD’s indicator database provide a great deal of data relevant to HRH planning in member countries, a more comprehensive approach consistent with the criteria outlined in the methodology section would require additional information as well as direct engagement with the relevant stakeholder groups in the respective countries. As such, the methods used have several important limitations that must be considered when interpreting the results:

- Because none of the data included in these analyses were collected by the authors of this paper, we are not in a position to assess their accuracy.
- The need to rely on secondary descriptions of the included countries’ health systems means that a full understanding of their respective objectives may not have been achieved—in other words, criterion 1 may not have been fully met in all cases.
- Neither the OECD nor WHO databases distinguishes between HRH practicing in the private and public sectors. As such, these analyses do not distinguish between care provided in the public and private sectors.
- During the review of existing analyses of HRH requirements conducted as part of the first paper, no existing methods were found that explicitly incorporated determinants of productivity—i.e., that met criterion 3. Although such models have been described (e.g., [24, 25]), it was not possible to obtain the data necessary to incorporate this feature into the simulations. Instead, the sensitivity of the projections to different levels of productivity is demonstrated.
- Although the OECD indicator database includes information on average remuneration paid to general and specialist physicians as well as hospital nurses for many member countries, no multi-country source of information on training or recruitment costs was found. As a result, cost considerations are not incorporated into these simulations.
- As shown in Table 1, the information needed to perform simulations in accordance with the other criteria was not readily available in many cases. As such, a variety of assumptions were made in order to produce the simulations:
  - ◦For most countries, no information on how health care service provision is organized according to the objectives of their various health care systems—beyond improving the health of their respective populations—was found. In fact, only for Canada and Australia was information on service provision according to different levels of health found. For the other countries, current Canadian levels of service provision according to different levels of health were used to estimate requirements.
  - ◦The measure of health status available by age and sex for most included countries was self-assessed health status. This measure was used to simulate the requirements for nursing and physician services in each country.
    - ■This measure was available for all included countries except Chile. As such, simulations of the requirements for nurses and physicians in Chile could not be performed.
    - ■Although self-assessed health is a commonly used health outcome measure that has been found to be highly correlated with physician assessments [26, 27] and to be highly predictive of both health care utilization [28] and mortality [29, 30, 31] across a wide range of populations, there is also evidence that this measure may be subject to important limitations which would reduce its validity as a measure of need for health care. These include, for example, scale of reference bias (respondents may “adjust” their reported health status based on what they perceive to be the norm for those of similar age or circumstances) [32]. There may also be socioeconomic and/or cultural differences across countries in how such questions are answered (e.g., [33]). Moreover, self-assessed health does not, on its own, fully capture the need that individuals may have for the health care services required by these populations.
    - ■A more comprehensive approach to population needs-based planning would identify requirements by types of need/condition (e.g., [19, 20, 33]) and then aggregate over all needs/conditions. Although other measures such as the incidence or prevalence of specific health problems (e.g., human immunodeficiency virus/acquired immunodeficiency syndrome (HIV/AIDS), diabetes) are available for virtually every country, these are seldom presented by age and sex of patient. More complex measures such as disability-adjusted life years (DALYs) lost to poor health are also widely available at the country level and could potentially be used to prioritize population health issues so that health care resources (including HRH) can be allocated accordingly. However, these measures are not meant to provide measures of health care need at the individual level; we know of no clinical guidelines for the treatment of DALYs, for example. Similarly, mortality data are also widely available but are not as useful for prospective HRH planning as individual-level measures of health status.
  - ◦Data on the incidence of low birth weight in the included countries are available from the OECD indicator database. However, these data are not available by the age of mothers; as such, it was assumed that the incidence of low birth weight was equal across mother age groups within countries. For countries whose female populations are aging, this would likely result in underestimates of the numbers of babies born underweight, while for countries whose female populations are becoming younger, this would likely result in overestimates of the numbers of babies born underweight.
- In the absence of “gold standards” defining appropriate levels of health care service provision by age, sex, and health status, the values included in the model are based on current values. This is done for the purposes of demonstrating the model’s application and does not imply that these levels are optimal relative to the objectives of each country’s health care systems—for example, they may not reflect the service levels required to meet population health needs. Planners within individual countries can and should update these data (and any others they desire) to reflect planned levels of service provision within their respective jurisdictions. To illustrate this functionality, the impact of different values for the level of service parameter is shown in the “Discussion” section.
  - ◦The measures of service provision found for most countries—physician consultations, nights in hospital, and numbers of births—were not presented by level of acuity nor do they fully capture the wide range of services provided by midwives, nurses, or physicians. However, they were the only measures of service provision found for most countries. As such, these relatively crude measures of service provision—and hence the productivity of the different professions—were used as proxies of overall service provision to simulate requirements.
  - ◦As information on the proportion of pregnancies and births attended by midwives (as opposed to physicians, for example) was not available across countries, it was assumed that these proportions for each country—whatever they may currently be—would be maintained throughout the simulation period.
  - ◦As information on unmet need for health care was not found for most countries, the estimates are initialized using an initial HRH “gap” of zero. Hence, the surpluses or shortfalls simulated represent changes to any existing imbalance between supply and requirements in each country. For cases where an existing shortfall or surplus has been documented and quantified, the model can be initialized at any value desired.
- Both the WHO and OECD indicator databases provide relatively recent (usually from 2011 or later for the WHO database and 2013 or later for the OECD database) head counts of midwives, nurses, and physicians for most member countries with the notable exception of Greece, for which no information on the supply of midwives or nurses since 2005 was found. In the absence of any recent information on the current supply of these professionals, simulations of the future supply of midwives and nurses in Greece could not be performed.
- Midwives, nurses, and physicians have different qualifications and functions in different countries; the country-specific analyses conducted for this paper are therefore based on whatever definitions of these professions were used by the reporting countries in submitting data to the OECD and WHO.
- Although the OECD and WHO indicator databases provide separate counts of nurses and midwives, it is possible that some reporting countries may have counted individuals as members of both professions when submitting this data. In these cases the supply of both professions would be overestimated.
  - ◦The OECD indicator database also provides breakdowns of countries’ physician supplies by age. However, age breakdowns were not available for midwives or nurses from any multi-country source. For some countries, age distributions of midwives and nurses were found in individual documents obtained as part of preparing the first paper. For other countries, in the absence of such information, the age distribution observed for those providers in Canada was used to illustrate the application of the model.
- The OECD indicator database provides historical data on annual numbers of graduates for various health professions in most of the included countries. No information on numbers of midwifery graduates in Portugal was found; it was assumed that no new graduates from that profession in that country would join its supply during the simulation period. If in fact there are midwifery graduates being trained in Portugal, this assumption would bias the simulations toward underestimating its future midwife supply.
  - ◦No multi-country source of information on the retention of new HRH graduates was found. As such, it was assumed that all graduates of different professions in individual countries would enter their respective countries’ supplies. This assumption would likely bias the simulations toward overestimating future HRH supply.
- The OECD indicator database also includes historical data on physician and nurse migration for most member countries. It does not include information on migration of midwives. For countries where no information on in-migrants of a particular profession was found, it was assumed that no new members of that profession would join the respective stocks of these provider groups during the study period. This assumption would likely bias the simulations toward underestimating future HRH supply in these cases.
- No multi-country source of information on retirements or other exits from the respective national supplies was found. For some countries, this information was provided in documents obtained as part of Phase 1, but for countries and professions for which this information was not found, the simulations were run based on an assumption that 5% of the existing stock would exit each year.
- For most member countries, the OECD indicator database differentiates between the numbers of midwives and nurses licensed to practice and the numbers actively practicing; for these countries, this information provided the basis for estimating levels of participation by these professions. For the few countries where this information was not available, the average participation level of the other countries was used.
  - ◦The OECD indicator database does not provide comparable participation information for physicians nor does any other multi-country source found. For some countries, information on physician participation levels was found in individual documents obtained as part of Phase 1. In the absence of this information, it was assumed that all licensed physicians are active in providing at least some direct patient care. This assumption would likely bias the simulations toward overestimating the future HRH supply in these cases.
- No multi-country data source found includes information on levels of activity (e.g., average hours worked per week) by HRH. For some countries, this information was found in individual documents obtained as part of Phase 1. For countries where such information was not found, it was assumed that all members of these professions work full-time hours. This assumption would likely bias the simulations toward overestimating the future HRH supply in these cases.

In the absence of reliable data on the future values of various model variables, all except the size and age-sex distribution of the population were held constant for the production of the baseline simulations. However, univariate and multivariate sensitivity analyses were conducted modulating the assumptions on each individual model parameter, as well as on several parameters simultaneously, to explore and illustrate how the results produced can change according to different scenarios. These analyses were based on actual variation in parameter values found within one of the included countries. Thus, the baseline simulations should not be interpreted as predictions of what will happen; instead, they are meant to serve as compass bearings, showing the directions in which the HRH situations in the included countries are heading, and what policy levers may be used to move toward a better matching of HRH supply with population needs.

## Results

The supply of and requirements for midwives, nurses, and physicians in each included country were simulated for the period up to 2030, together with the surplus or shortfall implied by these results. The simulated surpluses or shortfalls for each profession and country are provided in Table 2. These simulation results suggest that, if the current HRH situations in the included countries continue to 2030, most of the included countries could face shortfalls of one or more types of HRH; that is, holding constant all the parameters included in the model except population, they would not have enough HRH available to continue to provide their current levels of health care services to their respective populations. In contrast, some countries may experience surpluses of some types of HRH; that is, they would have more than the number needed to continue to provide current levels of health care services to their respective populations. In total, these simulations in the baseline scenarios sum to shortfalls of over 45 000 midwives, 1.1 million nurses, and 754 000 physicians across the 31 included countries for 2030. These simulated shortfalls are the collective result of simulated future supplies of 157 000 midwives, 6.8 million nurses, and 2.4 million physicians against simulated requirements of 202 000 midwives, 7.9 million nurses, and 3.2 million physicians.

**Table 2:** Simulated HRH shortfalls or surpluses by profession and country for 2030—based on demographic change alone

Country	Profession	Projected shortfall (−) or surplus (+)	Country	Profession	Projected shortfall (−) or surplus (+)
Australia	Midwives	−9 068	Italy	Midwives	−1 779
	Nurses	−144 654		Nurses	−153 147
	Physicians	−23 393		Physicians	−89 467
Austria	Midwives	−238	Japan	Midwives	−7 086
	Nurses	+13 505		Nurses	+58 188
	Physicians	−12 584		Physicians	−7 579
Belgium	Midwives	−1 428	Luxembourg	Midwives	−112
	Nurses	−65 590		Nurses	−5 156
	Physicians	−5 365		Physicians	−1 252
Canada	Midwives	−437	Netherlands	Midwives	−773
	Nurses	−84 719		Nurses	−31 068
	Physicians	+8 108		Physicians	−8 369
Chile	Midwives	−4 735	New Zealand	Midwives	+1 271
	Nurses			Nurses	−10 035
	Physicians			Physicians	+5 297
Czech Republic	Midwives	−544	Norway	Midwives	−1 192
	Nurses	−31 975		Nurses	−47 886
	Physicians	−9 195		Physicians	−13 057
Denmark	Midwives	−202	Poland	Midwives	+2 042
	Nurses	−46 540		Nurses	−48 036
	Physicians	−475		Physicians	−16 524
Estonia	Midwives	+70	Portugal	Midwives	−752
	Nurses	−6 319		Nurses	−8 538
	Physicians	−3 375		Physicians	−13 285
Finland	Midwives	+622	Slovakia	Midwives	−126
	Nurses	−1 361		Nurses	+8 304
	Physicians	−956		Physicians	−5 238
France	Midwives	−3 485	Slovenia	Midwives	+180
	Nurses	−177 497		Nurses	+13 626
	Physicians	−83 950		Physicians	−82
Germany	Midwives	−2 058	South Korea	Midwives	−474
	Nurses	−135 236		Nurses	+104 127
	Physicians	−73 130		Physicians	−27 861
Greece	Midwives		Spain	Midwives	+54
	Nurses			Nurses	−85 858
	Physicians	−33 761		Physicians	−27 341
Hungary	Midwives	−510	Sweden	Midwives	−3 576
	Nurses	+11 027		Nurses	−57 574
	Physicians	−4 398		Physicians	−9 352
Iceland	Midwives	−174	Switzerland	Midwives	−262
	Nurses	−1 294		Nurses	−4 690
	Physicians	−499		Physicians	−14 581
Ireland	Midwives	−5 618	United Kingdom	Midwives	−2 225
	Nurses	−32 498		Nurses	−36 240
	Physicians	+7 026		Physicians	+33 318
Israel	Midwives	−781	United States	Midwives	−2 630
	Nurses	−24 181		Nurses	−143 302
	Physicians	−23 789		Physicians	−303 760

These simulations are not to be taken as predictions about the future. Should large shortfalls or surpluses appear on the planning horizon, it is likely that substantial changes to these variables—such as increases or reductions in training capacity or levels of service provision—will be made; indeed the purpose of performing these simulations is to inform such planning. The shortfalls or surpluses shown here are measured relative to any shortfalls or surpluses that may already exist for these professions in these countries. In these simulations, all other planning parameters are held constant. Analyses demonstrating the sensitivity of these simulations to changes in other planning parameters are provided elsewhere in this report.

## Discussion

It is important to note that, despite the large amount of data used to generate them, these results are based on numerous assumptions. For some countries, the assumptions described above may be very close to reality; for others, they may be quite different. With access to more accurate and comprehensive country-specific information, planners in individual countries can easily replace these assumptions with relevant data to better reflect their actual HRH situations. Further, despite the above limitations, considerable effort was put into estimating the current or “baseline” value of the various parameters used to simulate HRH supply and requirements for physicians, nurses, and midwives for the included countries. However, it is not possible to accurately predict what values these parameters will take on in the future. As such, these results should not be interpreted as predictions of how the HRH situations in the included countries will change through 2030 but rather what may happen if each of the many assumptions used in producing them are accurate from the present until that time. It is, of course, exceedingly unlikely that this is will be the case; in fact, the values of each planning parameter are likely to be somehow different than has been assumed in producing these results.

### Sensitivity analyses

To demonstrate the sensitivity of these simulations to different future variable values, as well as the capacity of this approach to accommodate these, several different scenarios pertaining to the future values of key model parameters are presented below. Although providing such detailed sensitivity analyses for each of the three professions and country would require a prohibitively wide array of empirical data, analyses for one profession in one country—physicians in Canada—are provided below. Canadian physicians were chosen as the demonstration case because that was the only country and profession for which a sufficient range of empirical data on which to base such scenarios were found.

**Fig. 2:**
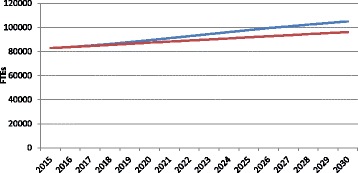
Example simulated physician supply and requirements to 2030. *Blue* supply, *Red* requirements

**Fig. 3:**
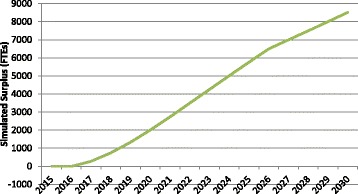
Example simulated physician gap to 2030

As a frame of reference for the other analyses, Fig. 2 shows the simulated supply of and requirements for physicians through 2030, measured in FTEs, under a scenario in which all planning variables except population are held constant at current levels. In this simulation, both the supply of and requirements for physicians in Canada increase through 2030, with supply increasing at a more rapid rate, resulting in a growing surplus (notwithstanding any existing gap) of just under 9000 FTEs by 2030. The difference or “gap” between the simulated supply of and requirements for physicians in Canada is shown in more detail in Fig. 3.

#### Impacts of changes in HRH productivity

**Fig. 4:**
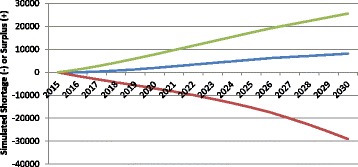
Examples of simulated physician gap in different productivity scenarios. *Green* productivity increases, *Blue* productivity remains constant, *Red* productivity decreases

In the context of HRH planning, the term productivity refers to the number and type of services full-time equivalent HRH on average can be reasonably expected to provide at some basic standard of quality. For the purposes of these simulations, HRH productivity is measured in terms of numbers of consultations for physicians, hospital patient days for nurses, and births for midwives. There are substantial differences in the values of these measures across and even within countries and jurisdictions. Figure 4 shows the impact of different assumed values for productivity on the estimated physician gap for Canada. Each of the three curves begins with a productivity value set at the average number of consultations per FTE physician across the country as captured in administrative data collected by the Canadian Institute for Health Information [34]. The middle or “baseline” curve shows how the simulated physician gap would change if this level of productivity remained at the current national average level through 2030. The lower of the three curves represents the simulated gap should productivity decrease to the lowest currently found among Canadian provinces, while the upper curve shows how the estimates would change should productivity increase to the level found in the province with the highest average numbers of consultations per physician in the country.

Figure 4 demonstrates the sensitivity of HRH estimates to different levels of productivity: if all Canadian physicians became, on average, as productive as those in the province with the highest reported productivity, this would result in a growing physician surplus by 2030, other things equal. Alternatively, should Canadian physicians become, on average, as productive as those in the province with the lowest reported productivity levels, this would result in a growing physician shortfall of even larger magnitude over the same time period, other things equal. The difference between these scenarios is approximately 55 000 FTEs, which would represent 70% the country’s current physician supply.

#### Impacts of changes in levels of service

**Fig. 5:**
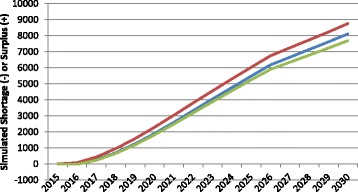
Examples of simulated physician gap in different level of service scenarios. *Red* levels of service increase, *Blue* levels of service remain constant, *Green* levels of service decrease

Figure 5 shows the impact of different assumed future levels of service provision on the simulated physician gap in Canada. Levels of service, in this context, refers to the average number of physician consultations to be provided per person, given the person’s age, sex, and health status. The higher the level of service—i.e., the greater the number of physician consultations to be provided—the more physicians are required to deliver those services, other things equal. In the “average service levels” scenario, the required number of physicians is based on the average number of physician consultations by patient age, sex, and health status across the country in 2012 based on data included in the 2012 iteration of Statistics Canada’s Canadian Community Health Survey [35]. In the “low service levels” scenario, physician requirements are simulated based on the average numbers of consultations reported by patients in the Canadian province whose residents report receiving the fewest physician consultations per person given their age, sex, and health status in the country. In the “high service levels” scenario, requirements are based on the average numbers of consultations per patient in the province where the average number of reported physician consultations given their age, sex, and health status is the highest in the country.

As Fig. 5 shows, should the level of physician services in 2030 be lower than it is currently—that is, should fewer physician consultations be required for patients of a given age, sex, and health status—the surplus of physicians would be higher than it would otherwise be. Similarly, should more physician consultations be required for a given level of health status by 2030, more physicians would be required to provide those consultations and the surplus would be lower than otherwise. The difference in the assumed future levels of service provision equates to a difference in the simulated physician gap of over 1000 FTEs by 2030.

#### Impacts of changes in health status

**Fig. 6:**
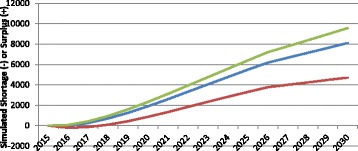
Examples of simulated physician gap in different health status scenarios. *Green* age-specific levels of health improve, *Blue* age-specific levels of health remain constant, *Red* age-specific levels of health worsen

The health status of the population to be served can also affect the requirements for HRH, since sicker people require more health care services, other things equal. Figure 6 illustrates examples of how changes in population health over time can impact the simulated “gap” in physicians.

The middle of the three curves shows the simulated physician gap (again, in this case, a surplus) for Canada if the current average level of health across the country remains the same through 2030. The other two curves show the impact on the gap of worsening or improving health status. More specifically, the latter scenario shows the trajectory of the estimated physician gap should the average age- and sex-specific self-reported health status of Canadians improve to match that of the province with the best self-reported health status. There are substantial differences in various measures of health status across Canada [36]. Similarly, the former shows the trajectory of the estimated physician gap should the average age- and sex-specific health status of Canadians worsen to match that of the province with the worst self-reported health status. The breakdowns of self-assessed health status by age, sex, and province were performed by the authors using data from the 2012 iteration of Statistics Canada’s Canadian Community Health Survey [35]. The difference between assumed future levels of health status in these scenarios translates into a difference in the projected physician gap of over 5000 FTEs by 2030.

#### Impacts of demographic changes

**Fig. 7:**
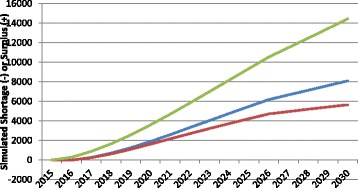
Examples of simulated physician gap in different population growth scenarios. *Green* low population growth, *Blue* median population growth, *Red* high population growth

An additional factor affecting simulations of requirements for health care services—and by extension the requirements for HRH—is the size and age-sex distribution of the population. Although considerable effort is put into developing estimates of the future population size and characteristics in many countries, predicting these features with accuracy remains difficult. Figure 7 shows the impact on the simulated Canadian physician gap of different assumed changes to population size. As Fig. 7 demonstrates, the trajectory of the simulated physician gap is substantially affected by the assumed demographic trajectory of the population those physicians are to serve. The difference between the high and low population growth scenarios—each of which is based on different projections developed by Statistics Canada [37]—results in a difference of over 10 000 FTEs in the simulated physician gap by 2030.

#### Impacts of changes in HRH activity

**Fig. 8:**
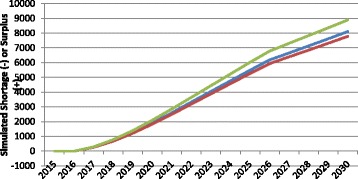
Examples of simulated physician gap in different physician activity scenarios. *Green* physician activity increases, *Blue* physician activity remains constant, *Red* physician activity decreases

The effective supply of HRH is dependent not only on the number of practicing members of various personnel but also on the time they collectively spend providing patient care. Figure 8 shows the impact of different assumed levels of physician activity on the simulated surplus.

The more time the average physician spends providing direct care to patients, the fewer physicians will be required, other things equal. In Canada, there is substantial variation in the hours that physicians report spending on direct patient care. The middle curve in Fig. 8 shows the trajectory of the projected physician surplus assuming the average Canadian physician spends the same number of hours on direct patient care in 2030 as reported by the average Canadian physician in 2014—39.6 h per week, including direct care provided during on-call hours, according to self-reported data from the National Physician Survey [38], which was developed by the Canadian Medical Association in partnership with several other national health care stakeholder organizations. The upper curve shows how the estimated surplus would be greater if the average Canadian physician were to work as many hours as the average physician in the province with the highest reported number of hours spent providing direct patient care—43.5 per week. Similarly, the lower curve shows how the estimated surplus would be reduced if the average Canadian physician were to work as many hours as the average physician in the province with the lowest reported number of hours spent providing direct patient care—38.0 per week. The difference between these scenarios equates to a difference in the projected physician surplus of over 1000 FTEs by 2030.

#### Impacts of changes in HRH training

**Fig. 9:**
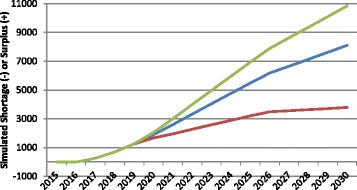
Examples of simulated physician gap in different physician graduation scenarios. *Green* physician graduation increases 15%, *Blue* physician graduation remains constant, *Red* physician graduation decreases 15%

In 2014, there were 2804 graduates of Canadian medical schools. This was the highest number of graduates in the country’s history and represents a 15% increase from the number of graduates in 2010 (2448). Figure 9 shows the impacts on the simulated physician gap of three different levels of annual physician graduation. In one, the level from 2014 is maintained through 2030. In the others, the number of graduates is decreased to the value from 2010 and increased another 15%.

Figure 9 illustrates the sensitivity of the simulated physician gap to different numbers of physician graduates. Although the changes take at least 4 years to have any impact (because of the duration of the training programs), these substantial differences in medical school graduates result in substantially different simulated future physician gap, with a simulated difference between the “low” and “high” graduation scenarios of roughly 7000 FTEs by 2030.

#### Impacts of changes in HRH participation

**Fig. 10:**
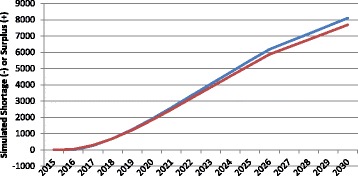
Examples of simulated physician gap in different physician participation scenarios. *Blue* physician participation remains at 95%, *Red* physician participation decreases to 90%

According to the most recent results of the annual National Physician Survey [38], an estimated 95% of physicians in Canada report providing at least some direct patient care, while the remaining 5% work exclusively in other roles such as administration or education. This is a higher level of participation than nurses in Canada, of whom 81% are actively practicing according to the OECD indicator database [21]. Figure 10 shows the impact of two different levels of participation on the simulated future physician gap in Canada: In one, the current level of 95% is maintained through 2030. In the other, it is decreased to 90%. As Fig. 10 illustrates, the simulated physician gap is directly affected by different assumed values of future participation levels, with the modest difference between 90 and 95% participation resulting in a difference of roughly 500 FTEs in the simulated gap by 2015.

#### Impacts of changes in HRH retention

**Fig. 11:**
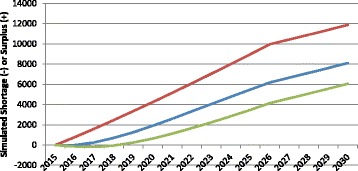
Examples of simulated physician gap in different exit rate scenarios. *Red* 2% older physician exit rates, *Blue* 7% older physician exit rates, *Green* 12% older physician exit rates

There is very high retention among physicians in Canada [39]; when physicians do exit the Canadian supply, it is usually due to migration, retirement, or death [40]. Canada is a net recipient of migrating physicians and has been for the past decade [41], and the most recent specific quantitative analysis of attrition among practicing physicians reported that less than 1% of physicians in Canada either die or retire before age 65 [42]. After age 65, the combined proportion of physicians retiring or dying each year increases with age; in recent years, this proportion has varied between 2 and 12% per year [42]. Figure 11 shows the impacts of three different exit rate scenarios on the simulated physician gap in Canada. In these, the proportion of physicians aged 65 and over exiting the Canadian supply each year is set at 2, 7, and 12%. The simulated physician gap is highly sensitive to different assumptions about the annual rates of exit from the supply. As Fig. 11 indicates, the difference between 2 and 12% of older physicians leaving per year is roughly 6000 FTEs by 2030.

#### Impacts of changes in multiple parameters

**Fig. 12:**
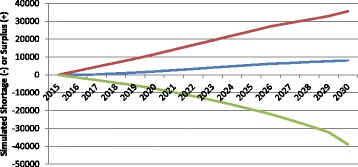
Cumulative impact of multiple parameter changes on simulated physician gap. *Red* low requirements, high supply; *Blue* status quo continues; *Green* high requirements, low supply

The scenarios above show the potential impacts of changes in individual planning variables over time. In reality, it is likely that more than one—and perhaps all—of the variables that affect estimated HRH supply and requirements will change to some degree between now and 2030. Figure 12 shows the potential cumulative impacts of each of the individual changes described in the earlier scenarios on the simulated physician gap in Canada in three scenarios. In the “status quo continues” scenario, no variables change except the population grows and ages according to Statistics Canada’s medium growth projections. In the “high requirements, low supply” scenario, the population grows and ages according to Statistics Canada’s “high growth” projections, the average age-specific health of Canadians worsens to that of its least healthy province, physician service levels increase to the highest in its provinces, exit rates among older physicians occur at the highest they have been in recent years, physician activity and productivity decrease to the lowest levels reported in its provinces, graduation is reduced to 2010 levels, and participation reduced to 90%. In the “low requirements, high supply” situation, the population grows and ages according to Statistics Canada’s “low growth” projections, the average age-specific health of Canadians improves to that of its healthiest province, physician service levels are reduced to the lowest found in its provinces, exit rates among older physicians occur at the lowest they have been in recent years, physician activity and productivity increase to the highest levels in its provinces, graduation is increased 15%, and participation remains at 95%.

Figure 12 demonstrates that, depending on which assumed values are assigned to the various variables that determine HRH supply and requirements over time, the simulated physician gap in Canada for 2030 varies considerably. In these examples, it may range anywhere from a shortfall of nearly 39 000 FTEs (47% less than the simulated supply) to a surplus of over 35 000 FTEs (43% greater than the simulated supply)—a difference of nearly 75 000 FTEs—depending on the assumptions made about the various factors that determine HRH supply and requirements.

If the same level of variability in the size of Canada’s physician gap relative to supply is applied to the aggregate gaps simulated for all 31 included countries, these could range from shortfalls of 74 000 midwives, 3.2 million nurses, and 1.2 million physicians (47% less than supply) to surpluses of 67 000 midwives, 2.9 million nurses, and 1.0 million physicians (43% greater than supply) by 2030.

### Implications

These results underscore the importance of several factors critical to effective HRH planning, each of which have been repeatedly identified by earlier analyses of HRH planning worldwide.

First, HRH planning must be conducted iteratively, recognizing the constantly evolving nature of population health needs, health care practices and regulatory structures, and health labor markets, and incorporating ongoing monitoring and evaluation. This is especially necessary in countries such as Australia, Canada, and the United Kingdom that include separate health care systems at the sub-national (e.g., state or provincial) levels. Long-term simulations such as those included here are useful for showing the direction in which HRH situations may be heading, but as the results presented here demonstrate, they cannot be used to predict the future. As such, HRH plans must be regularly updated to accommodate changes in planning variables over time (e.g., [2, 43–46]).

Second, HRH planning must be conducted by engaging with the relevant stakeholders (e.g., [43, 45, 47, 48]). This is necessary to ensure that the approach being used is relevant, that the information necessary to utilize a valid approach is made available, and that planning gives appropriate consideration to the perspectives of different stakeholder groups. Planners in individual countries would have direct access to these stakeholders, which was not possible for the analyses reported here.

Third, HRH planning must be based on appropriate data, and this includes measures of both the health levels of the population as well as the health care services planned to respond to those health levels, together with characteristics of the health workforce and the broader economic, socio-political, environmental, and technological contexts in which the planning is being done (e.g., [43, 47, 49–52]). The relatively crude measures used for the simulations in this report were chosen because they were either the only measures available or the only ones provided for multiple countries from a single source. Comprehensive analyses of HRH supply and requirements can and have been conducted to inform national-level HRH policies and strategies (e.g., [19, 20, 33]). These analyses can also be performed at the sub-national level, for example, in countries such as Canada [37] which are home to multiple distinct health care systems. It is important to note that the more comprehensive data required to support such analyses do in general exist in high-income countries, for example, the data collected for Canada, most of which is publicly available online, were much more complete than for other countries.

Other, more commonly used approaches to health workforce planning have been widely and repeatedly recognized as ineffective. Over 40 years ago, the WHO noted that

The exclusive use of health manpower: population ratios for the estimation of future health manpower requirements is increasingly difficult to justify in the developed countries where, depending on the specific characteristics of the health system, other and more refined criteria can be used to improve the country’s capacity to provide health care. [43]

Along similar lines, the UK House of Commons Health Committee [53] commented on the “disastrous failure of (health) workforce planning” which it attributes to the fact that “little if any thought has been given to long term or strategic planning.” A report commissioned in response called for workforce planning to “be based on service planning and …reflect how health and social care will meet the needs of the local population” [54]. More recently, the Global Strategy on Human Resources for Health recommended that all countries “Build planning capacity to develop or improve HRH policy and strategies that quantify health workforce needs, demands and supply under different future scenarios” [7]. Consistent with such calls, the strength of the workforce planning approach presented here lies in its use of methods that are linked directly to service planning based on the needs of populations but that can at the same time accommodate the uncertainty—and the opportunity for policy intervention—in relation to a range of health systems and health workforce parameters. The results of such approaches cannot be meaningfully compared to those generated by more commonly used workforce planning models which cannot account for differences in populations’ needs for care or the levels of services required to meet those needs. The approaches may produce very different results because they are addressing very different research or health policy questions implicit in the planning models being used.

There were numerous data-related challenges encountered through this study that hindered efforts to simulate future HRH supply and requirements, most especially the latter. Perhaps related to this problem, the review and synthesis of recent analyses of HRH requirements in the included countries conducted as the first phase of this work found that most of these focused primarily on measures of HRH supply and utilization. This is perhaps because these are more readily available, which may in turn be at least partially due to the lack of priority given to the collection of data pertaining to HRH requirements (in addition to HRH supply) in documents such as the WHO’s recommended minimum HRH dataset [59]. The WHO’s global strategy on HRH for 2030, in contrast, outlines multiple planned activities to increase health systems’ capacity to assess and respond to the health needs of their respective populations through improved HRH data collection and planning. It also makes several recommendations that would, if acted upon, help to address several of the limitations of the HRH supply data available for this study, for example, it specifies that HRH supply should be measured in FTEs as opposed to head counts [7].

However, as noted in the first paper, problems with data are not avoided by relying on more conceptually limited models. If the information available is inadequate to fully inform planning, then investments should be made in improving the quality of that information rather than in further entrenching the use of intrinsically narrower models. To that end, the identification and assessment of the data required to inform HRH planning should be based on the question of how many of what type of HRH are required to perform what services, for whom, and under what circumstances (e.g., [20, 47, 55]). The HRH planning approach described in this report and its companion paper are designed to address this question and to overcome the limitations of more traditional approaches which have resulted in the numerous HRH challenges being experienced worldwide.

While the focus of this analysis was on midwives, nurses, and physicians in high-income OECD countries, the analytical approach described can theoretically be applied in any jurisdiction or profession for which the required data are available. Similar approaches have already been applied, for example, to maternal health teams in Guinea [56], pharmacists in Jamaica [57], dental care teams in Thailand [58], and HIV/AIDS and malaria teams Zambia [59].

## Conclusions

The objective of this paper was to simulate the supply of and requirements for midwives, nurses, and physicians in high-income OECD countries through 2030, to inform global policy dialog and strategic planning at both international and national levels, such as that occurring as part of the development of the global strategy on HRH. This was carried out using publicly available data and using assumptions about the values of the various determinants of supply and requirements, aside from population projections, remaining constant throughout that time period. Despite the limitations resulting from challenges obtaining some of the necessary data in the time available, the results demonstrate the potential to apply an approach to simulating HRH supply and requirements in a manner more comprehensive than most of those currently being used in many countries. HRH planners in individual countries, who have more direct access to data on the relevant planning parameters, are best placed to implement such an approach working with their respective stakeholder groups.

The results of these simulations suggest that the trajectories of HRH supplies and requirements vary widely across countries and professions, with some potentially headed for large surpluses, while others may be moving toward large shortfalls. These findings reinforce the concern highlighted by the WHO in its Global Strategy on Human Resources for Health: Workforce 2030 [7] that many high-income countries are challenged with matching supply and requirements for HRH under existing and future affordability and sustainability constraints and often experience periodic swings between perceived shortfalls and oversupply. These can be particularly problematic from both domestic and international perspectives; for example, domestic underproduction and/or maldistribution of health workers can contribute to a disproportionate reliance on recruitment of foreign-trained health personnel. Historically, the included countries as a group have been the beneficiaries of the bulk of international migration of health workers from low- and middle-income countries, worsening deficits in these contexts, and that these mobility trends are likely to continue and even increase in the future [60]. In this context, the analysis reported here provides evidence that can also inform a global policy dialog on international imbalances and mobility patterns across countries. The type of HRH planning approach described here also allows the identification of the policy levers with the greatest potential to affect trends and prevent the emergence of future gaps, thereby informing policy options that may alleviate or avoid these gaps, such as changes to models of care delivery, HRH training, and/or regulatory structures.

The sensitivity analyses demonstrate that these simulations are highly dependent on the assumptions on current and future values of the various variables that determine HRH supply and requirements. These findings highlight the importance of HRH planning that is iterative, based on a valid methodology, and engages the relevant stakeholder groups. Further, it is imperative that these stakeholders collaborate to ensure that the types of data necessary for such methodologies are regularly collected and analyzed to inform HRH planning. Evidence-based analyses of HRH situations require reliable and regularly updated information on population health status, labor market dynamics, and health service configurations.

## Additional files

Additional file 1: Stated objectives of included countries’ health care systems. (DOCX 18 kb)
Additional file 2: Data sources by country. (DOCX 87 kb)

## Abbreviations

AIDS: Acquired immunodeficiency syndrome
DALYs: Disability-adjusted life years
EU: European Union
GHWA: Global Health Workforce Alliance
HIV: Human immunodeficiency virus
HRH: Human resources for health
OECD: Organisation for Economic Co-operation and Development
PAHO: Pan American Health Organization
RN: Registered nurse
WHO: World Health Organization

## Footnotes

1 Latvia is now a high-income country and member of the OECD, but did not become the latter until after these analyses were conducted.
