# Supplementary material for: Simulating future supply of and requirements for human resources for health in high-income OECD countries
Source: Hum Resour Health. 2016 Dec 12;14:77. doi: 10.1186/s12960-016-0168-x (PMC5154072; doi:10.1186/s12960-016-0168-x)
Supplement: Additional file 1: — Stated objectives of included countries’ health care systems. (DOCX 18 kb) [file 12960_2016_168_MOESM1_ESM.docx]

Appendix I

Stated Objectives of Included Countries’ Health Care Systems

| Country | Equity | Access | Maintain- Improve Health | Service According to Needs | Quality | Efficiency | Sustainability | Comprehensive | Universal | Patient Choice | Portability | Public Funding | PHC | Transparency | Patient-Centred |
| --- | --- | --- | --- | --- | --- | --- | --- | --- | --- | --- | --- | --- | --- | --- | --- |
| Australia | • | • | • | • | • | • | • |  | • |  |  |  | • |  |  |
| Austria | • | • |  | • | • | • |  | • | • |  |  |  |  |  |  |
| Belgium |  | • |  |  | • |  | • |  |  |  |  |  |  |  |  |
| Canada |  |  |  | • |  |  |  | • | • |  | • | • |  |  |  |
| Chile | • |  |  |  | • |  |  |  | • |  |  |  |  |  |  |
| Czech Rep. | • | • |  |  |  |  | • |  | • |  |  |  |  |  |  |
| Denmark | • | • | • | • | • |  |  |  | • | • |  |  |  | • |  |
| Estonia |  |  |  |  | • |  | • |  |  | • |  |  | • |  | • |
| Finland | • | • | • |  |  |  |  |  |  |  |  |  | • |  |  |
| France | • | • |  |  |  |  |  | • | • | • |  |  |  |  |  |
| Germany |  | • | • | • | • | • |  |  |  |  |  |  |  |  |  |
| Greece | • |  |  |  | • |  |  | • | • |  |  |  |  |  |  |
| Iceland | • | • | • | • | • | • |  |  |  |  |  |  |  |  |  |
| Ireland |  | • | • |  |  |  |  |  |  |  |  |  | • |  |  |
| Israel | • |  | • |  |  |  |  |  |  |  |  |  |  |  |  |
| Italy | • | • |  |  |  | • |  | • | • |  | • | • |  |  |  |
| Japan |  |  |  |  | • |  |  |  |  |  |  |  |  |  |  |
| Luxembourg | • | • | • |  | • |  |  |  |  |  |  |  | • |  |  |
| Netherlands | • | • |  |  |  |  |  |  |  |  |  |  |  |  |  |
| New Zealand | • | • | • |  |  |  | • |  | • |  |  | • | • |  |  |
| Norway | • |  |  | • | • | • |  |  | • |  |  |  |  |  |  |
| Poland | • | • | • | • |  |  |  |  |  |  |  | • |  |  |  |
| Portugal | • | • | • |  | • |  |  |  |  |  |  |  |  |  |  |
| Slovakia | • | • |  |  |  |  |  |  | • |  |  |  |  |  |  |
| Slovenia |  |  |  | • |  |  | • |  |  |  |  |  |  |  |  |
| South Korea | • | • | • |  | • | • |  |  | • |  |  |  |  |  |  |
| Spain |  | • |  |  |  |  |  |  | • |  |  | • | • |  |  |
| Sweden | • | • | • | • |  | • |  |  |  |  |  |  |  |  |  |
| Switzerland | • |  | • |  | • |  |  |  |  |  |  |  |  | • |  |
| UK | • | • | • | • | • |  |  | • |  |  |  | • |  | • |  |
| USA | • |  | • |  |  |  |  |  |  |  |  |  |  |  |  |
