# Supplementary material for: Simulating future supply of and requirements for human resources for health in high-income OECD countries
Source: Hum Resour Health. 2016 Dec 12;14:77. doi: 10.1186/s12960-016-0168-x (PMC5154072; doi:10.1186/s12960-016-0168-x)
Supplement: Additional file 2: — Data sources by country. (DOCX 87 kb) [file 12960_2016_168_MOESM2_ESM.docx]

**Australia**

| **Planning Parameter** | **Data Source** | | |
| --- | --- | --- | --- |
| *Population characteristics* |  | | |
| Population size and projections by age and sex | OECD Stat | | |
| Health status by age and sex | OECD Stat | | |
| *Health care system characteristics* | **Midwives Model** | **Nurses Model** | **Physicians Model** |
| Health care provision by age, sex, and health status | N/A | Australia Health Survey^[[1]](#footnote-1)^ | Australia Health Survey |
| Service provision per FTE provider per year | OECD Stat | OECD Stat | OECD Stat |
| Average total annual wages per FTE | N/A | N/A | N/A |
| Average training cost per graduate | N/A | N/A | N/A |
| Average replacement cost per FTE | N/A | N/A | N/A |
| Number of new graduates per year | OECD Stat | OECD Stat | OECD Stat |
| Age distribution of new graduates | N/A | N/A | N/A |
| Number of in-migrants per year | Health Workforce Australia (HWA)^[[2]](#footnote-2)^241/as be participation levels New Zealandion in that country would join its supply during the simulation periodnd, it was as | OECD Stat | OECD Stat |
| Age distribution of in-migrants | N/A | N/A | N/A |
| Exits per year by age | HWA | HWA | HWA |
| Total yearly enrolment in training programs | N/A | N/A | N/A |
| % of students completing training | N/A | N/A | N/A |
| % of graduates remaining in-jurisdiction | N/A | N/A | N/A |
| Head count of current supply | OECD Stat | OECD Stat | OECD Stat |
| Age distribution of current supply | HWA | HWA | OECD Stat |
| % of licensed workforce providing any patient care | OECD Stat | OECD Stat | OECD Stat |
| Average hours worked/week by participating workforce | N/A | N/A | N/A |

**Austria**

| **Planning Parameter** | **Data Source** | | |
| --- | --- | --- | --- |
| *Population characteristics* |  | | |
| Population size and projections by age and sex | OECD Stat | | |
| Health status by age and sex | OECD Stat | | |
| *Health care system characteristics* | **Midwives Model** | **Nurses Model** | **Physicians Model** |
| Health care provision by age, sex, and health status | N/A | N/A | N/A |
| Service provision per FTE provider per year | OECD Stat | OECD Stat | OECD Stat |
| Average total annual wages per FTE | N/A | N/A | OECD Stat |
| Average training cost per graduate | N/A | N/A | N/A |
| Average replacement cost per FTE | N/A | N/A | N/A |
| Number of new graduates per year | OECD Stat | OECD Stat | OECD Stat |
| Age distribution of new graduates | N/A | N/A | N/A |
| Number of in-migrants per year | N/A241/as be participation levels New Zealandion in that country would join its supply during the simulation periodnd, it was as | OECD Stat | OECD Stat |
| Age distribution of in-migrants | N/A | N/A | N/A |
| Exits per year by age | N/A | N/A | N/A |
| Total yearly enrolment in training programs | N/A | N/A | N/A |
| % of students completing training | N/A | N/A | N/A |
| % of graduates remaining in-jurisdiction | N/A | N/A | N/A |
| Head count of current supply | OECD Stat | OECD Stat | OECD Stat |
| Age distribution of current supply | N/A | N/A | OECD Stat |
| % of licensed workforce providing any patient care | OECD Stat | OECD Stat | OECD Stat |
| Average hours worked/week by participating workforce | N/A | N/A | N/A |

**Belgium**

| **Planning Parameter** | **Data Source** | | |
| --- | --- | --- | --- |
| *Population characteristics* |  | | |
| Population size and projections by age and sex | OECD Stat | | |
| Health status by age and sex | OECD Stat | | |
| *Health care system characteristics* | **Midwives Model** | **Nurses Model** | **Physicians Model** |
| Health care provision by age, sex, and health status | N/A | N/A | N/A |
| Service provision per FTE provider per year | OECD Stat | OECD Stat | OECD Stat |
| Average total annual wages per FTE | N/A | OECD Stat | N/A |
| Average training cost per graduate | N/A | N/A | N/A |
| Average replacement cost per FTE | N/A | N/A | N/A |
| Number of new graduates per year | OECD Stat | OECD Stat | OECD Stat |
| Age distribution of new graduates | N/A | N/A | N/A |
| Number of in-migrants per year | N/A241/as be participation levels New Zealandion in that country would join its supply during the simulation periodnd, it was as | OECD Stat | OECD Stat |
| Age distribution of in-migrants | N/A | N/A | N/A |
| Exits per year by age | N/A | N/A | N/A |
| Total yearly enrolment in training programs | N/A | N/A | N/A |
| % of students completing training | N/A | N/A | N/A |
| % of graduates remaining in-jurisdiction | N/A | N/A | N/A |
| Head count of current supply | OECD Stat | OECD Stat | OECD Stat |
| Age distribution of current supply | N/A | N/A | OECD Stat |
| % of licensed workforce providing any patient care | N/A | OECD Stat | OECD Stat |
| Average hours worked/week by participating workforce | N/A | N/A | N/A |

**Canada**

| **Planning Parameter** | **Data Source** | | |
| --- | --- | --- | --- |
| *Population characteristics* |  | | |
| Population size and projections by age and sex | OECD Stat | | |
| Health status by age and sex | OECD Stat | | |
| *Health care system characteristics* | **Midwives Model** | **Nurses Model** | **Physicians Model** |
| Health care provision by age, sex, and health status | N/A | CCHS^[[3]](#footnote-3)^ | CCHS |
| Service provision per FTE provider per year | OECD Stat | OECD Stat | NPDB^[[4]](#footnote-4)^ |
| Average total annual wages per FTE | N/A | OECD Stat | NPDB |
| Average training cost per graduate | N/A | N/A | N/A |
| Average replacement cost per FTE | N/A | O’Brien-Pallas et al., 2012^[[5]](#footnote-5)^ | N/A |
| Number of new graduates per year | OECD Stat | OECD Stat | OECD Stat |
| Age distribution of new graduates | N/A | Tomblin Murphy et al., 2012^[[6]](#footnote-6)^ | CAPER^[[7]](#footnote-7)^ |
| Number of in-migrants per year | N/A241/as be participation levels New Zealandion in that country would join its supply during the simulation periodnd, it was as | OECD Stat | OECD Stat |
| Age distribution of in-migrants | N/A | Tomblin Murphy et al., 2012 | CAPER |
| Exits per year by age | N/A | CIHI Health Workforce Database^[[8]](#footnote-8)^ | Pong, 2011^[[9]](#footnote-9)^ |
| Total yearly enrolment in training programs | N/A | Tomblin Murphy et al., 2012 | CMA^[[10]](#footnote-10)^ |
| % of students completing training | N/A | Tomblin Murphy et al., 2012 | CMA |
| % of graduates remaining in-jurisdiction | Canadian Association of Midwives (CAM)^[[11]](#footnote-11)^ | CIHI Health Workforce Database | CAPER |
| Head count of current supply | OECD Stat | OECD Stat | OECD Stat |
| Age distribution of current supply | N/A | CIHI Health Workforce Database | OECD Stat |
| % of licensed workforce providing any patient care | CAM | OECD Stat | OECD Stat |
| Average hours worked/week by participating workforce | N/A | Tomblin Murphy et al., 2012 | National Physician Survey^[[12]](#footnote-12)^ |

**Chile**

| **Planning Parameter** | **Data Source** | | |
| --- | --- | --- | --- |
| *Population characteristics* |  | | |
| Population size and projections by age and sex | OECD Stat | | |
| Health status by age and sex | N/A | | |
| *Health care system characteristics* | **Midwives Model** | **Nurses Model** | **Physicians Model** |
| Health care provision by age, sex, and health status | N/A | N/A | N/A |
| Service provision per FTE provider per year | N/A | N/A | N/A |
| Average total annual wages per FTE | N/A | OECD Stat | OECD Stat |
| Average training cost per graduate | N/A | N/A | N/A |
| Average replacement cost per FTE | N/A | N/A | N/A |
| Number of new graduates per year | OECD Stat | OECD Stat | OECD Stat |
| Age distribution of new graduates | N/A | N/A | N/A |
| Number of in-migrants per year | N/A241/as be participation levels New Zealandion in that country would join its supply during the simulation periodnd, it was as | N/A | N/A |
| Age distribution of in-migrants | N/A | N/A | N/A |
| Exits per year by age | N/A | N/A | N/A |
| Total yearly enrolment in training programs | N/A | N/A | N/A |
| % of students completing training | N/A | N/A | N/A |
| % of graduates remaining in-jurisdiction | N/A | N/A | N/A |
| Head count of current supply | N/A | N/A | N/A |
| Age distribution of current supply | N/A | N/A | N/A |
| % of licensed workforce providing any patient care | N/A | N/A | N/A |
| Average hours worked/week by participating workforce | N/A | N/A | N/A |

**Czech Republic**

| **Planning Parameter** | **Data Source** | | |
| --- | --- | --- | --- |
| *Population characteristics* |  | | |
| Population size and projections by age and sex | OECD Stat | | |
| Health status by age and sex | OECD Stat | | |
| *Health care system characteristics* | **Midwives Model** | **Nurses Model** | **Physicians Model** |
| Health care provision by age, sex, and health status | N/A | N/A | N/A |
| Service provision per FTE provider per year | OECD Stat | OECD Stat | OECD Stat |
| Average total annual wages per FTE | N/A | OECD Stat | N/A |
| Average training cost per graduate | N/A | N/A | N/A |
| Average replacement cost per FTE | N/A | N/A | N/A |
| Number of new graduates per year | OECD Stat | OECD Stat | OECD Stat |
| Age distribution of new graduates | N/A | N/A | N/A |
| Number of in-migrants per year | N/A241/as be participation levels New Zealandion in that country would join its supply during the simulation periodnd, it was as | N/A | OECD Stat |
| Age distribution of in-migrants | N/A | N/A | N/A |
| Exits per year by age | N/A | N/A | N/A |
| Total yearly enrolment in training programs | N/A | N/A | N/A |
| % of students completing training | N/A | N/A | N/A |
| % of graduates remaining in-jurisdiction | N/A | N/A | N/A |
| Head count of current supply | OECD Stat | OECD Stat | OECD Stat |
| Age distribution of current supply | N/A | N/A | OECD Stat |
| % of licensed workforce providing any patient care | OECD Stat | OECD Stat | OECD Stat |
| Average hours worked/week by participating workforce | N/A | N/A | N/A |

**Denmark**

| **Planning Parameter** | **Data Source** | | |
| --- | --- | --- | --- |
| *Population characteristics* |  | | |
| Population size and projections by age and sex | OECD Stat | | |
| Health status by age and sex | OECD Stat | | |
| *Health care system characteristics* | **Midwives Model** | **Nurses Model** | **Physicians Model** |
| Health care provision by age, sex, and health status | N/A | N/A | N/A |
| Service provision per FTE provider per year | OECD Stat | OECD Stat | OECD Stat |
| Average total annual wages per FTE | N/A | OECD Stat | N/A |
| Average training cost per graduate | N/A | N/A | N/A |
| Average replacement cost per FTE | N/A | N/A | N/A |
| Number of new graduates per year | OECD Stat | OECD Stat | OECD Stat |
| Age distribution of new graduates | N/A | N/A | N/A |
| Number of in-migrants per year | N/A241/as be participation levels New Zealandion in that country would join its supply during the simulation periodnd, it was as | OECD Stat | OECD Stat |
| Age distribution of in-migrants | N/A | N/A | N/A |
| Exits per year by age | N/A | N/A | N/A |
| Total yearly enrolment in training programs | N/A | N/A | N/A |
| % of students completing training | N/A | N/A | N/A |
| % of graduates remaining in-jurisdiction | N/A | N/A | N/A |
| Head count of current supply | OECD Stat | OECD Stat | OECD Stat |
| Age distribution of current supply | N/A | N/A | OECD Stat |
| % of licensed workforce providing any patient care | OECD Stat | OECD Stat | OECD Stat |
| Average hours worked/week by participating workforce | N/A | N/A | N/A |

**Estonia**

| **Planning Parameter** | **Data Source** | | |
| --- | --- | --- | --- |
| *Population characteristics* |  | | |
| Population size and projections by age and sex | OECD Stat | | |
| Health status by age and sex | OECD Stat | | |
| *Health care system characteristics* | **Midwives Model** | **Nurses Model** | **Physicians Model** |
| Health care provision by age, sex, and health status | N/A | N/A | N/A |
| Service provision per FTE provider per year | OECD Stat | OECD Stat | OECD Stat |
| Average total annual wages per FTE | N/A | OECD Stat | OECD Stat |
| Average training cost per graduate | N/A | N/A | N/A |
| Average replacement cost per FTE | N/A | N/A | N/A |
| Number of new graduates per year | OECD Stat | OECD Stat | OECD Stat |
| Age distribution of new graduates | N/A | N/A | N/A |
| Number of in-migrants per year | N/A241/as be participation levels New Zealandion in that country would join its supply during the simulation periodnd, it was as | OECD Stat | OECD Stat |
| Age distribution of in-migrants | N/A | N/A | N/A |
| Exits per year by age | N/A | N/A | N/A |
| Total yearly enrolment in training programs | N/A | N/A | N/A |
| % of students completing training | N/A | N/A | N/A |
| % of graduates remaining in-jurisdiction | N/A | N/A | N/A |
| Head count of current supply | OECD Stat | OECD Stat | OECD Stat |
| Age distribution of current supply | N/A | N/A | OECD Stat |
| % of licensed workforce providing any patient care | OECD Stat | OECD Stat | OECD Stat |
| Average hours worked/week by participating workforce | N/A | N/A | N/A |

**Finland**

| **Planning Parameter** | **Data Source** | | |
| --- | --- | --- | --- |
| *Population characteristics* |  | | |
| Population size and projections by age and sex | OECD Stat | | |
| Health status by age and sex | OECD Stat | | |
| *Health care system characteristics* | **Midwives Model** | **Nurses Model** | **Physicians Model** |
| Health care provision by age, sex, and health status | N/A | N/A | N/A |
| Service provision per FTE provider per year | OECD Stat | OECD Stat | OECD Stat |
| Average total annual wages per FTE | N/A | OECD Stat | OECD Stat |
| Average training cost per graduate | N/A | N/A | N/A |
| Average replacement cost per FTE | N/A | N/A | N/A |
| Number of new graduates per year | OECD Stat | OECD Stat | OECD Stat |
| Age distribution of new graduates | N/A | N/A | N/A |
| Number of in-migrants per year | N/A241/as be participation levels New Zealandion in that country would join its supply during the simulation periodnd, it was as | OECD Stat | OECD Stat |
| Age distribution of in-migrants | N/A | N/A | N/A |
| Exits per year by age | N/A | N/A | N/A |
| Total yearly enrolment in training programs | N/A | N/A | N/A |
| % of students completing training | N/A | N/A | N/A |
| % of graduates remaining in-jurisdiction | N/A | N/A | N/A |
| Head count of current supply | OECD Stat | OECD Stat | OECD Stat |
| Age distribution of current supply | N/A | N/A | OECD Stat |
| % of licensed workforce providing any patient care | OECD Stat | OECD Stat | OECD Stat |
| Average hours worked/week by participating workforce | N/A | N/A | N/A |

**France**

| **Planning Parameter** | **Data Source** | | |
| --- | --- | --- | --- |
| *Population characteristics* |  | | |
| Population size and projections by age and sex | OECD Stat | | |
| Health status by age and sex | OECD Stat | | |
| *Health care system characteristics* | **Midwives Model** | **Nurses Model** | **Physicians Model** |
| Health care provision by age, sex, and health status | N/A | N/A | N/A |
| Service provision per FTE provider per year | OECD Stat | OECD Stat | OECD Stat |
| Average total annual wages per FTE | N/A | OECD Stat | N/A |
| Average training cost per graduate | N/A | N/A | N/A |
| Average replacement cost per FTE | N/A | N/A | N/A |
| Number of new graduates per year | OECD Stat | OECD Stat | OECD Stat |
| Age distribution of new graduates | N/A | N/A | N/A |
| Number of in-migrants per year | N/A241/as be participation levels New Zealandion in that country would join its supply during the simulation periodnd, it was as | OECD Stat | OECD Stat |
| Age distribution of in-migrants | N/A | N/A | N/A |
| Exits per year by age | N/A | N/A | N/A |
| Total yearly enrolment in training programs | N/A | N/A | N/A |
| % of students completing training | N/A | N/A | N/A |
| % of graduates remaining in-jurisdiction | N/A | N/A | N/A |
| Head count of current supply | OECD Stat | OECD Stat | OECD Stat |
| Age distribution of current supply | N/A | N/A | OECD Stat |
| % of licensed workforce providing any patient care | OECD Stat | OECD Stat | OECD Stat |
| Average hours worked/week by participating workforce | N/A | N/A | N/A |

**Germany**

| **Planning Parameter** | **Data Source** | | |
| --- | --- | --- | --- |
| *Population characteristics* |  | | |
| Population size and projections by age and sex | OECD Stat | | |
| Health status by age and sex | OECD Stat | | |
| *Health care system characteristics* | **Midwives Model** | **Nurses Model** | **Physicians Model** |
| Health care provision by age, sex, and health status | N/A | N/A | N/A |
| Service provision per FTE provider per year | OECD Stat | OECD Stat | OECD Stat |
| Average total annual wages per FTE | N/A | N/A | N/A |
| Average training cost per graduate | N/A | N/A | N/A |
| Average replacement cost per FTE | N/A | N/A | N/A |
| Number of new graduates per year | OECD Stat | OECD Stat | OECD Stat |
| Age distribution of new graduates | N/A | N/A | N/A |
| Number of in-migrants per year | N/A241/as be participation levels New Zealandion in that country would join its supply during the simulation periodnd, it was as | OECD Stat | OECD Stat |
| Age distribution of in-migrants | N/A | N/A | N/A |
| Exits per year by age | N/A | N/A | N/A |
| Total yearly enrolment in training programs | N/A | N/A | N/A |
| % of students completing training | N/A | N/A | N/A |
| % of graduates remaining in-jurisdiction | N/A | N/A | N/A |
| Head count of current supply | OECD Stat | OECD Stat | OECD Stat |
| Age distribution of current supply | N/A | N/A | OECD Stat |
| % of licensed workforce providing any patient care | OECD Stat | OECD Stat | OECD Stat |
| Average hours worked/week by participating workforce | N/A | N/A | N/A |

**Greece**

| **Planning Parameter** | **Data Source** | | |
| --- | --- | --- | --- |
| *Population characteristics* |  | | |
| Population size and projections by age and sex | OECD Stat | | |
| Health status by age and sex | OECD Stat | | |
| *Health care system characteristics* | **Midwives Model** | **Nurses Model** | **Physicians Model** |
| Health care provision by age, sex, and health status | N/A | N/A | N/A |
| Service provision per FTE provider per year | OECD Stat | OECD Stat | OECD Stat |
| Average total annual wages per FTE | N/A | N/A | N/A |
| Average training cost per graduate | N/A | N/A | N/A |
| Average replacement cost per FTE | N/A | N/A | N/A |
| Number of new graduates per year | OECD Stat | OECD Stat | OECD Stat |
| Age distribution of new graduates | N/A | N/A | N/A |
| Number of in-migrants per year | N/A241/as be participation levels New Zealandion in that country would join its supply during the simulation periodnd, it was as | OECD Stat | OECD Stat |
| Age distribution of in-migrants | N/A | N/A | N/A |
| Exits per year by age | N/A | N/A | N/A |
| Total yearly enrolment in training programs | N/A | N/A | N/A |
| % of students completing training | N/A | N/A | N/A |
| % of graduates remaining in-jurisdiction | N/A | N/A | N/A |
| Head count of current supply | OECD Stat | OECD Stat | N/A |
| Age distribution of current supply | N/A | N/A | N/A |
| % of licensed workforce providing any patient care | OECD Stat | OECD Stat | N/A |
| Average hours worked/week by participating workforce | N/A | N/A | N/A |

**Hungary**

| **Planning Parameter** | **Data Source** | | |
| --- | --- | --- | --- |
| *Population characteristics* |  | | |
| Population size and projections by age and sex | OECD Stat | | |
| Health status by age and sex | OECD Stat | | |
| *Health care system characteristics* | **Midwives Model** | **Nurses Model** | **Physicians Model** |
| Health care provision by age, sex, and health status | N/A | N/A | N/A |
| Service provision per FTE provider per year | OECD Stat | OECD Stat | OECD Stat |
| Average total annual wages per FTE | N/A | OECD Stat | OECD Stat |
| Average training cost per graduate | N/A | N/A | N/A |
| Average replacement cost per FTE | N/A | N/A | N/A |
| Number of new graduates per year | OECD Stat | OECD Stat | OECD Stat |
| Age distribution of new graduates | N/A | N/A | N/A |
| Number of in-migrants per year | N/A241/as be participation levels New Zealandion in that country would join its supply during the simulation periodnd, it was as | OECD Stat | OECD Stat |
| Age distribution of in-migrants | N/A | N/A | N/A |
| Exits per year by age | N/A | N/A | N/A |
| Total yearly enrolment in training programs | N/A | N/A | N/A |
| % of students completing training | N/A | N/A | N/A |
| % of graduates remaining in-jurisdiction | N/A | N/A | N/A |
| Head count of current supply | OECD Stat | OECD Stat | OECD Stat |
| Age distribution of current supply | N/A | N/A | OECD Stat |
| % of licensed workforce providing any patient care | OECD Stat | OECD Stat | OECD Stat |
| Average hours worked/week by participating workforce | N/A | N/A | N/A |

**Iceland**

| **Planning Parameter** | **Data Source** | | |
| --- | --- | --- | --- |
| *Population characteristics* |  | | |
| Population size and projections by age and sex | OECD Stat | | |
| Health status by age and sex | OECD Stat | | |
| *Health care system characteristics* | **Midwives Model** | **Nurses Model** | **Physicians Model** |
| Health care provision by age, sex, and health status | N/A | N/A | N/A |
| Service provision per FTE provider per year | OECD Stat | OECD Stat | OECD Stat |
| Average total annual wages per FTE | N/A | OECD Stat | OECD Stat |
| Average training cost per graduate | N/A | N/A | N/A |
| Average replacement cost per FTE | N/A | N/A | N/A |
| Number of new graduates per year | OECD Stat | OECD Stat | OECD Stat |
| Age distribution of new graduates | N/A | N/A | N/A |
| Number of in-migrants per year | N/A241/as be participation levels New Zealandion in that country would join its supply during the simulation periodnd, it was as | N/A | N/A |
| Age distribution of in-migrants | N/A | N/A | N/A |
| Exits per year by age | N/A | N/A | N/A |
| Total yearly enrolment in training programs | N/A | N/A | N/A |
| % of students completing training | N/A | N/A | N/A |
| % of graduates remaining in-jurisdiction | N/A | N/A | N/A |
| Head count of current supply | OECD Stat | OECD Stat | OECD Stat |
| Age distribution of current supply | N/A | N/A | OECD Stat |
| % of licensed workforce providing any patient care | OECD Stat | OECD Stat | OECD Stat |
| Average hours worked/week by participating workforce | N/A | N/A | N/A |

**Ireland**

| **Planning Parameter** | **Data Source** | | |
| --- | --- | --- | --- |
| *Population characteristics* |  | | |
| Population size and projections by age and sex | OECD Stat | | |
| Health status by age and sex | OECD Stat | | |
| *Health care system characteristics* | **Midwives Model** | **Nurses Model** | **Physicians Model** |
| Health care provision by age, sex, and health status | N/A | N/A | N/A |
| Service provision per FTE provider per year | OECD Stat | OECD Stat | OECD Stat |
| Average total annual wages per FTE | N/A | OECD Stat | N/A |
| Average training cost per graduate | N/A | N/A | N/A |
| Average replacement cost per FTE | N/A | N/A | N/A |
| Number of new graduates per year | OECD Stat | OECD Stat | OECD Stat |
| Age distribution of new graduates | N/A | N/A | N/A |
| Number of in-migrants per year | N/A241/as be participation levels New Zealandion in that country would join its supply during the simulation periodnd, it was as | OECD Stat | OECD Stat |
| Age distribution of in-migrants | N/A | N/A | N/A |
| Exits per year by age | N/A | N/A | N/A |
| Total yearly enrolment in training programs | N/A | N/A | N/A |
| % of students completing training | N/A | N/A | N/A |
| % of graduates remaining in-jurisdiction | N/A | N/A | N/A |
| Head count of current supply | OECD Stat | OECD Stat | OECD Stat |
| Age distribution of current supply | N/A | N/A | OECD Stat |
| % of licensed workforce providing any patient care | N/A | OECD Stat | OECD Stat |
| Average hours worked/week by participating workforce | N/A | N/A | N/A |

**Israel**

| **Planning Parameter** | **Data Source** | | |
| --- | --- | --- | --- |
| *Population characteristics* |  | | |
| Population size and projections by age and sex | OECD Stat | | |
| Health status by age and sex | OECD Stat | | |
| *Health care system characteristics* | **Midwives Model** | **Nurses Model** | **Physicians Model** |
| Health care provision by age, sex, and health status | N/A | N/A | N/A |
| Service provision per FTE provider per year | OECD Stat | OECD Stat | OECD Stat |
| Average total annual wages per FTE | N/A | OECD Stat | OECD Stat |
| Average training cost per graduate | N/A | N/A | N/A |
| Average replacement cost per FTE | N/A | N/A | N/A |
| Number of new graduates per year | OECD Stat | OECD Stat | OECD Stat |
| Age distribution of new graduates | N/A | N/A | N/A |
| Number of in-migrants per year | N/A241/as be participation levels New Zealandion in that country would join its supply during the simulation periodnd, it was as | OECD Stat | OECD Stat |
| Age distribution of in-migrants | N/A | N/A | N/A |
| Exits per year by age | N/A | N/A | N/A |
| Total yearly enrolment in training programs | N/A | N/A | N/A |
| % of students completing training | N/A | N/A | N/A |
| % of graduates remaining in-jurisdiction | N/A | N/A | N/A |
| Head count of current supply | OECD Stat | OECD Stat | OECD Stat |
| Age distribution of current supply | N/A | N/A | OECD Stat |
| % of licensed workforce providing any patient care | N/A | OECD Stat | OECD Stat |
| Average hours worked/week by participating workforce | N/A | N/A | N/A |

**Italy**

| **Planning Parameter** | **Data Source** | | |
| --- | --- | --- | --- |
| *Population characteristics* |  | | |
| Population size and projections by age and sex | OECD Stat | | |
| Health status by age and sex | OECD Stat | | |
| *Health care system characteristics* | **Midwives Model** | **Nurses Model** | **Physicians Model** |
| Health care provision by age, sex, and health status | N/A | N/A | N/A |
| Service provision per FTE provider per year | OECD Stat | OECD Stat | OECD Stat |
| Average total annual wages per FTE | N/A | OECD Stat | N/A |
| Average training cost per graduate | N/A | N/A | N/A |
| Average replacement cost per FTE | N/A | N/A | N/A |
| Number of new graduates per year | OECD Stat | OECD Stat | OECD Stat |
| Age distribution of new graduates | N/A | N/A | N/A |
| Number of in-migrants per year | N/A241/as be participation levels New Zealandion in that country would join its supply during the simulation periodnd, it was as | OECD Stat | OECD Stat |
| Age distribution of in-migrants | N/A | N/A | N/A |
| Exits per year by age | N/A | N/A | N/A |
| Total yearly enrolment in training programs | N/A | N/A | N/A |
| % of students completing training | N/A | N/A | N/A |
| % of graduates remaining in-jurisdiction | N/A | N/A | N/A |
| Head count of current supply | OECD Stat | OECD Stat | OECD Stat |
| Age distribution of current supply | N/A | N/A | OECD Stat |
| % of licensed workforce providing any patient care | N/A | N/A | OECD Stat |
| Average hours worked/week by participating workforce | N/A | N/A | N/A |

**Japan**

| **Planning Parameter** | **Data Source** | | |
| --- | --- | --- | --- |
| *Population characteristics* |  | | |
| Population size and projections by age and sex | OECD Stat | | |
| Health status by age and sex | OECD Stat | | |
| *Health care system characteristics* | **Midwives Model** | **Nurses Model** | **Physicians Model** |
| Health care provision by age, sex, and health status | N/A | N/A | N/A |
| Service provision per FTE provider per year | OECD Stat | OECD Stat | OECD Stat |
| Average total annual wages per FTE | N/A | OECD Stat | N/A |
| Average training cost per graduate | N/A | N/A | N/A |
| Average replacement cost per FTE | N/A | N/A | N/A |
| Number of new graduates per year | OECD Stat | OECD Stat | OECD Stat |
| Age distribution of new graduates | N/A | N/A | N/A |
| Number of in-migrants per year | N/A241/as be participation levels New Zealandion in that country would join its supply during the simulation periodnd, it was as | N/A | N/A |
| Age distribution of in-migrants | N/A | N/A | N/A |
| Exits per year by age | N/A | N/A | N/A |
| Total yearly enrolment in training programs | N/A | N/A | N/A |
| % of students completing training | N/A | N/A | N/A |
| % of graduates remaining in-jurisdiction | N/A | N/A | N/A |
| Head count of current supply | OECD Stat | OECD Stat | OECD Stat |
| Age distribution of current supply | N/A | N/A | OECD Stat |
| % of licensed workforce providing any patient care | OECD Stat | OECD Stat | OECD Stat |
| Average hours worked/week by participating workforce | N/A | N/A | N/A |

**Luxembourg**

| **Planning Parameter** | **Data Source** | | |
| --- | --- | --- | --- |
| *Population characteristics* |  | | |
| Population size and projections by age and sex | OECD Stat | | |
| Health status by age and sex | OECD Stat | | |
| *Health care system characteristics* | **Midwives Model** | **Nurses Model** | **Physicians Model** |
| Health care provision by age, sex, and health status | N/A | N/A | N/A |
| Service provision per FTE provider per year | OECD Stat | OECD Stat | OECD Stat |
| Average total annual wages per FTE | N/A | OECD Stat | OECD Stat |
| Average training cost per graduate | N/A | N/A | N/A |
| Average replacement cost per FTE | N/A | N/A | N/A |
| Number of new graduates per year | OECD Stat | OECD Stat | OECD Stat |
| Age distribution of new graduates | N/A | N/A | N/A |
| Number of in-migrants per year | N/A241/as be participation levels New Zealandion in that country would join its supply during the simulation periodnd, it was as | N/A | N/A |
| Age distribution of in-migrants | N/A | N/A | N/A |
| Exits per year by age | N/A | N/A | N/A |
| Total yearly enrolment in training programs | N/A | N/A | N/A |
| % of students completing training | N/A | N/A | N/A |
| % of graduates remaining in-jurisdiction | N/A | N/A | N/A |
| Head count of current supply | OECD Stat | OECD Stat | OECD Stat |
| Age distribution of current supply | N/A | N/A | OECD Stat |
| % of licensed workforce providing any patient care | OECD Stat | OECD Stat | OECD Stat |
| Average hours worked/week by participating workforce | N/A | N/A | N/A |

**Netherlands**

| **Planning Parameter** | **Data Source** | | |
| --- | --- | --- | --- |
| *Population characteristics* |  | | |
| Population size and projections by age and sex | OECD Stat | | |
| Health status by age and sex | OECD Stat | | |
| *Health care system characteristics* | **Midwives Model** | **Nurses Model** | **Physicians Model** |
| Health care provision by age, sex, and health status | N/A | N/A | N/A |
| Service provision per FTE provider per year | OECD Stat | OECD Stat | OECD Stat |
| Average total annual wages per FTE | N/A | OECD Stat | OECD Stat |
| Average training cost per graduate | N/A | N/A | N/A |
| Average replacement cost per FTE | N/A | N/A | N/A |
| Number of new graduates per year | OECD Stat | OECD Stat | OECD Stat |
| Age distribution of new graduates | N/A | N/A | N/A |
| Number of in-migrants per year | N/A241/as be participation levels New Zealandion in that country would join its supply during the simulation periodnd, it was as | N/A | N/A |
| Age distribution of in-migrants | N/A | N/A | N/A |
| Exits per year by age | N/A | N/A | N/A |
| Total yearly enrolment in training programs | N/A | N/A | N/A |
| % of students completing training | N/A | N/A | N/A |
| % of graduates remaining in-jurisdiction | N/A | N/A | N/A |
| Head count of current supply | OECD Stat | OECD Stat | OECD Stat |
| Age distribution of current supply | N/A | N/A | OECD Stat |
| % of licensed workforce providing any patient care | OECD Stat | OECD Stat | N/A |
| Average hours worked/week by participating workforce | N/A | N/A | N/A |

**New Zealand**

| **Planning Parameter** | **Data Source** | | |
| --- | --- | --- | --- |
| *Population characteristics* |  | | |
| Population size and projections by age and sex | OECD Stat | | |
| Health status by age and sex | OECD Stat | | |
| *Health care system characteristics* | **Midwives Model** | **Nurses Model** | **Physicians Model** |
| Health care provision by age, sex, and health status | N/A | N/A | N/A |
| Service provision per FTE provider per year | OECD Stat | OECD Stat | OECD Stat |
| Average total annual wages per FTE | N/A | OECD Stat | OECD Stat |
| Average training cost per graduate | N/A | N/A | N/A |
| Average replacement cost per FTE | N/A | N/A | N/A |
| Number of new graduates per year | OECD Stat | OECD Stat | OECD Stat |
| Age distribution of new graduates | N/A | N/A | N/A |
| Number of in-migrants per year | N/A241/as be participation levels New Zealandion in that country would join its supply during the simulation periodnd, it was as | OECD Stat | OECD Stat |
| Age distribution of in-migrants | N/A | N/A | N/A |
| Exits per year by age | N/A | N/A | N/A |
| Total yearly enrolment in training programs | N/A | N/A | N/A |
| % of students completing training | N/A | N/A | N/A |
| % of graduates remaining in-jurisdiction | N/A | N/A | N/A |
| Head count of current supply | OECD Stat | OECD Stat | OECD Stat |
| Age distribution of current supply | N/A | N/A | OECD Stat |
| % of licensed workforce providing any patient care | OECD Stat | OECD Stat | OECD Stat |
| Average hours worked/week by participating workforce | N/A | N/A | N/A |

**Norway**

| **Planning Parameter** | **Data Source** | | |
| --- | --- | --- | --- |
| *Population characteristics* |  | | |
| Population size and projections by age and sex | OECD Stat | | |
| Health status by age and sex | OECD Stat | | |
| *Health care system characteristics* | **Midwives Model** | **Nurses Model** | **Physicians Model** |
| Health care provision by age, sex, and health status | N/A | N/A | N/A |
| Service provision per FTE provider per year | OECD Stat | OECD Stat | OECD Stat |
| Average total annual wages per FTE | N/A | OECD Stat | N/A |
| Average training cost per graduate | N/A | N/A | N/A |
| Average replacement cost per FTE | N/A | N/A | N/A |
| Number of new graduates per year | OECD Stat | OECD Stat | OECD Stat |
| Age distribution of new graduates | N/A | N/A | N/A |
| Number of in-migrants per year | N/A241/as be participation levels New Zealandion in that country would join its supply during the simulation periodnd, it was as | OECD Stat | OECD Stat |
| Age distribution of in-migrants | N/A | N/A | N/A |
| Exits per year by age | N/A | N/A | N/A |
| Total yearly enrolment in training programs | N/A | N/A | N/A |
| % of students completing training | N/A | N/A | N/A |
| % of graduates remaining in-jurisdiction | N/A | N/A | N/A |
| Head count of current supply | OECD Stat | OECD Stat | OECD Stat |
| Age distribution of current supply | N/A | N/A | OECD Stat |
| % of licensed workforce providing any patient care | OECD Stat | OECD Stat | OECD Stat |
| Average hours worked/week by participating workforce | N/A | N/A | N/A |

**Poland**

| **Planning Parameter** | **Data Source** | | |
| --- | --- | --- | --- |
| *Population characteristics* |  | | |
| Population size and projections by age and sex | OECD Stat | | |
| Health status by age and sex | OECD Stat | | |
| *Health care system characteristics* | **Midwives Model** | **Nurses Model** | **Physicians Model** |
| Health care provision by age, sex, and health status | N/A | N/A | N/A |
| Service provision per FTE provider per year | OECD Stat | OECD Stat | OECD Stat |
| Average total annual wages per FTE | N/A | OECD Stat | OECD Stat |
| Average training cost per graduate | N/A | N/A | N/A |
| Average replacement cost per FTE | N/A | N/A | N/A |
| Number of new graduates per year | OECD Stat | OECD Stat | OECD Stat |
| Age distribution of new graduates | N/A | N/A | N/A |
| Number of in-migrants per year | N/A241/as be participation levels New Zealandion in that country would join its supply during the simulation periodnd, it was as | OECD Stat | OECD Stat |
| Age distribution of in-migrants | N/A | N/A | N/A |
| Exits per year by age | N/A | N/A | N/A |
| Total yearly enrolment in training programs | N/A | N/A | N/A |
| % of students completing training | N/A | N/A | N/A |
| % of graduates remaining in-jurisdiction | N/A | N/A | N/A |
| Head count of current supply | OECD Stat | OECD Stat | OECD Stat |
| Age distribution of current supply | N/A | N/A | OECD Stat |
| % of licensed workforce providing any patient care | OECD Stat | OECD Stat | OECD Stat |
| Average hours worked/week by participating workforce | N/A | N/A | N/A |

**Portugal**

| **Planning Parameter** | **Data Source** | | |
| --- | --- | --- | --- |
| *Population characteristics* |  | | |
| Population size and projections by age and sex | OECD Stat | | |
| Health status by age and sex | OECD Stat | | |
| *Health care system characteristics* | **Midwives Model** | **Nurses Model** | **Physicians Model** |
| Health care provision by age, sex, and health status | N/A | N/A | N/A |
| Service provision per FTE provider per year | OECD Stat | OECD Stat | OECD Stat |
| Average total annual wages per FTE | N/A | N/A | N/A |
| Average training cost per graduate | N/A | N/A | N/A |
| Average replacement cost per FTE | N/A | N/A | N/A |
| Number of new graduates per year | N/A | OECD Stat | OECD Stat |
| Age distribution of new graduates | N/A | N/A | N/A |
| Number of in-migrants per year | N/A241/as be participation levels New Zealandion in that country would join its supply during the simulation periodnd, it was as | N/A | N/A |
| Age distribution of in-migrants | N/A | N/A | N/A |
| Exits per year by age | N/A | N/A | N/A |
| Total yearly enrolment in training programs | N/A | N/A | N/A |
| % of students completing training | N/A | N/A | N/A |
| % of graduates remaining in-jurisdiction | N/A | N/A | N/A |
| Head count of current supply | OECD Stat | OECD Stat | OECD Stat |
| Age distribution of current supply | N/A | N/A | OECD Stat |
| % of licensed workforce providing any patient care | N/A | N/A | N/A |
| Average hours worked/week by participating workforce | N/A | N/A | N/A |

**Slovakia**

| **Planning Parameter** | **Data Source** | | |
| --- | --- | --- | --- |
| *Population characteristics* |  | | |
| Population size and projections by age and sex | OECD Stat | | |
| Health status by age and sex | OECD Stat | | |
| *Health care system characteristics* | **Midwives Model** | **Nurses Model** | **Physicians Model** |
| Health care provision by age, sex, and health status | N/A | N/A | N/A |
| Service provision per FTE provider per year | OECD Stat | OECD Stat | OECD Stat |
| Average total annual wages per FTE | N/A | OECD Stat | N/A |
| Average training cost per graduate | N/A | N/A | N/A |
| Average replacement cost per FTE | N/A | N/A | N/A |
| Number of new graduates per year | N/A | OECD Stat | OECD Stat |
| Age distribution of new graduates | N/A | N/A | N/A |
| Number of in-migrants per year | N/A241/as be participation levels New Zealandion in that country would join its supply during the simulation periodnd, it was as | N/A | N/A |
| Age distribution of in-migrants | N/A | N/A | N/A |
| Exits per year by age | N/A | N/A | N/A |
| Total yearly enrolment in training programs | N/A | N/A | N/A |
| % of students completing training | N/A | N/A | N/A |
| % of graduates remaining in-jurisdiction | N/A | N/A | N/A |
| Head count of current supply | OECD Stat | OECD Stat | OECD Stat |
| Age distribution of current supply | N/A | N/A | OECD Stat |
| % of licensed workforce providing any patient care | N/A | N/A | N/A |
| Average hours worked/week by participating workforce | N/A | N/A | N/A |

**Slovenia**

| **Planning Parameter** | **Data Source** | | |
| --- | --- | --- | --- |
| *Population characteristics* |  | | |
| Population size and projections by age and sex | OECD Stat | | |
| Health status by age and sex | OECD Stat | | |
| *Health care system characteristics* | **Midwives Model** | **Nurses Model** | **Physicians Model** |
| Health care provision by age, sex, and health status | N/A | N/A | N/A |
| Service provision per FTE provider per year | OECD Stat | OECD Stat | OECD Stat |
| Average total annual wages per FTE | N/A | OECD Stat | OECD Stat |
| Average training cost per graduate | N/A | N/A | N/A |
| Average replacement cost per FTE | N/A | N/A | N/A |
| Number of new graduates per year | N/A | OECD Stat | OECD Stat |
| Age distribution of new graduates | N/A | N/A | N/A |
| Number of in-migrants per year | N/A241/as be participation levels New Zealandion in that country would join its supply during the simulation periodnd, it was as | N/A | N/A |
| Age distribution of in-migrants | N/A | N/A | N/A |
| Exits per year by age | N/A | N/A | N/A |
| Total yearly enrolment in training programs | N/A | N/A | N/A |
| % of students completing training | N/A | N/A | N/A |
| % of graduates remaining in-jurisdiction | N/A | N/A | N/A |
| Head count of current supply | OECD Stat | OECD Stat | OECD Stat |
| Age distribution of current supply | N/A | N/A | OECD Stat |
| % of licensed workforce providing any patient care | OECD Stat | OECD Stat | OECD Stat |
| Average hours worked/week by participating workforce | N/A | N/A | N/A |

**South Korea**

| **Planning Parameter** | **Data Source** | | |
| --- | --- | --- | --- |
| *Population characteristics* |  | | |
| Population size and projections by age and sex | OECD Stat | | |
| Health status by age and sex | OECD Stat | | |
| *Health care system characteristics* | **Midwives Model** | **Nurses Model** | **Physicians Model** |
| Health care provision by age, sex, and health status | N/A | N/A | N/A |
| Service provision per FTE provider per year | OECD Stat | OECD Stat | OECD Stat |
| Average total annual wages per FTE | N/A | N/A | N/A |
| Average training cost per graduate | N/A | N/A | N/A |
| Average replacement cost per FTE | N/A | N/A | N/A |
| Number of new graduates per year | OECD Stat | OECD Stat | OECD Stat |
| Age distribution of new graduates | N/A | N/A | N/A |
| Number of in-migrants per year | N/A241/as be participation levels New Zealandion in that country would join its supply during the simulation periodnd, it was as | N/A | N/A |
| Age distribution of in-migrants | N/A | N/A | N/A |
| Exits per year by age | N/A | N/A | N/A |
| Total yearly enrolment in training programs | N/A | N/A | N/A |
| % of students completing training | N/A | N/A | N/A |
| % of graduates remaining in-jurisdiction | N/A | N/A | N/A |
| Head count of current supply | OECD Stat | OECD Stat | OECD Stat |
| Age distribution of current supply | N/A | N/A | OECD Stat |
| % of licensed workforce providing any patient care | OECD Stat | OECD Stat | OECD Stat |
| Average hours worked/week by participating workforce | N/A | N/A | N/A |

**Spain**

| **Planning Parameter** | **Data Source** | | |
| --- | --- | --- | --- |
| *Population characteristics* |  | | |
| Population size and projections by age and sex | OECD Stat | | |
| Health status by age and sex | OECD Stat | | |
| *Health care system characteristics* | **Midwives Model** | **Nurses Model** | **Physicians Model** |
| Health care provision by age, sex, and health status | N/A | N/A | N/A |
| Service provision per FTE provider per year | OECD Stat | OECD Stat | OECD Stat |
| Average total annual wages per FTE | N/A | OECD Stat | OECD Stat |
| Average training cost per graduate | N/A | N/A | N/A |
| Average replacement cost per FTE | N/A | N/A | N/A |
| Number of new graduates per year | N/A | OECD Stat | OECD Stat |
| Age distribution of new graduates | N/A | N/A | N/A |
| Number of in-migrants per year | N/A241/as be participation levels New Zealandion in that country would join its supply during the simulation periodnd, it was as | N/A | N/A |
| Age distribution of in-migrants | N/A | N/A | N/A |
| Exits per year by age | N/A | N/A | N/A |
| Total yearly enrolment in training programs | N/A | N/A | N/A |
| % of students completing training | N/A | N/A | N/A |
| % of graduates remaining in-jurisdiction | N/A | N/A | N/A |
| Head count of current supply | OECD Stat | OECD Stat | OECD Stat |
| Age distribution of current supply | N/A | N/A | OECD Stat |
| % of licensed workforce providing any patient care | N/A | N/A | OECD Stat |
| Average hours worked/week by participating workforce | N/A | N/A | N/A |

**Sweden**

| **Planning Parameter** | **Data Source** | | |
| --- | --- | --- | --- |
| *Population characteristics* |  | | |
| Population size and projections by age and sex | OECD Stat | | |
| Health status by age and sex | OECD Stat | | |
| *Health care system characteristics* | **Midwives Model** | **Nurses Model** | **Physicians Model** |
| Health care provision by age, sex, and health status | N/A | N/A | N/A |
| Service provision per FTE provider per year | OECD Stat | OECD Stat | OECD Stat |
| Average total annual wages per FTE | N/A | OECD Stat | OECD Stat |
| Average training cost per graduate | N/A | N/A | N/A |
| Average replacement cost per FTE | N/A | N/A | N/A |
| Number of new graduates per year | OECD Stat | OECD Stat | OECD Stat |
| Age distribution of new graduates | N/A | N/A | N/A |
| Number of in-migrants per year | N/A241/as be participation levels New Zealandion in that country would join its supply during the simulation periodnd, it was as | OECD Stat | OECD Stat |
| Age distribution of in-migrants | N/A | N/A | N/A |
| Exits per year by age | N/A | N/A | N/A |
| Total yearly enrolment in training programs | N/A | N/A | N/A |
| % of students completing training | N/A | N/A | N/A |
| % of graduates remaining in-jurisdiction | N/A | N/A | N/A |
| Head count of current supply | OECD Stat | OECD Stat | OECD Stat |
| Age distribution of current supply | N/A | N/A | OECD Stat |
| % of licensed workforce providing any patient care | OED Stat | OECD Stat | OECD Stat |
| Average hours worked/week by participating workforce | N/A | N/A | N/A |

**Switzerland**

| **Planning Parameter** | **Data Source** | | |
| --- | --- | --- | --- |
| *Population characteristics* |  | | |
| Population size and projections by age and sex | OECD Stat | | |
| Health status by age and sex | OECD Stat | | |
| *Health care system characteristics* | **Midwives Model** | **Nurses Model** | **Physicians Model** |
| Health care provision by age, sex, and health status | N/A | N/A | N/A |
| Service provision per FTE provider per year | OECD Stat | OECD Stat | OECD Stat |
| Average total annual wages per FTE | N/A | N/A | N/A |
| Average training cost per graduate | N/A | N/A | N/A |
| Average replacement cost per FTE | N/A | N/A | N/A |
| Number of new graduates per year | OECD Stat | OECD Stat | OECD Stat |
| Age distribution of new graduates | N/A | N/A | N/A |
| Number of in-migrants per year | N/A241/as be participation levels New Zealandion in that country would join its supply during the simulation periodnd, it was as | N/A | N/A |
| Age distribution of in-migrants | N/A | N/A | N/A |
| Exits per year by age | N/A | N/A | N/A |
| Total yearly enrolment in training programs | N/A | N/A | N/A |
| % of students completing training | N/A | N/A | N/A |
| % of graduates remaining in-jurisdiction | N/A | N/A | N/A |
| Head count of current supply | OECD Stat | OECD Stat | OECD Stat |
| Age distribution of current supply | N/A | N/A | OECD Stat |
| % of licensed workforce providing any patient care | OED Stat | OECD Stat | OECD Stat |
| Average hours worked/week by participating workforce | N/A | N/A | N/A |

**United States**

| **Planning Parameter** | **Data Source** | | |
| --- | --- | --- | --- |
| *Population characteristics* |  | | |
| Population size and projections by age and sex | OECD Stat | | |
| Health status by age and sex | OECD Stat | | |
| *Health care system characteristics* | **Midwives Model** | **Nurses Model** | **Physicians Model** |
| Health care provision by age, sex, and health status | N/A | N/A | N/A |
| Service provision per FTE provider per year | OECD Stat | OECD Stat | OECD Stat |
| Average total annual wages per FTE | N/A | N/A | N/A |
| Average training cost per graduate | N/A | N/A | N/A |
| Average replacement cost per FTE | N/A | N/A | N/A |
| Number of new graduates per year | OECD Stat | OECD Stat | OECD Stat |
| Age distribution of new graduates | N/A | N/A | N/A |
| Number of in-migrants per year | N/A241/as be participation levels New Zealandion in that country would join its supply during the simulation periodnd, it was as | N/A | N/A |
| Age distribution of in-migrants | N/A | N/A | N/A |
| Exits per year by age | N/A | HRSA, 2014^[[13]](#footnote-13)^ | N/A |
| Total yearly enrolment in training programs | N/A | N/A | N/A |
| % of students completing training | N/A | N/A | N/A |
| % of graduates remaining in-jurisdiction | N/A | N/A | N/A |
| Head count of current supply | OECD Stat | OECD Stat | OECD Stat |
| Age distribution of current supply | N/A | HRSA, 2013^[[14]](#footnote-14)^ | OECD Stat |
| % of licensed workforce providing any patient care | OED Stat | OECD Stat | OECD Stat |
| Average hours worked/week by participating workforce | N/A | HRSA, 2013 | N/A |

**United Kingdom**

| **Planning Parameter** | **Data Source** | | |
| --- | --- | --- | --- |
| *Population characteristics* |  | | |
| Population size and projections by age and sex | OECD Stat | | |
| Health status by age and sex | OECD Stat | | |
| *Health care system characteristics* | **Midwives Model** | **Nurses Model** | **Physicians Model** |
| Health care provision by age, sex, and health status | N/A | N/A | N/A |
| Service provision per FTE provider per year | OECD Stat | OECD Stat | OECD Stat |
| Average total annual wages per FTE | N/A | OECD Stat | OECD Stat |
| Average training cost per graduate | N/A | N/A | N/A |
| Average replacement cost per FTE | N/A | N/A | N/A |
| Number of new graduates per year | OECD Stat | OECD Stat | OECD Stat |
| Age distribution of new graduates | N/A | N/A | N/A |
| Number of in-migrants per year | N/A241/as be participation levels New Zealandion in that country would join its supply during the simulation periodnd, it was as | OECD Stat | OECD Stat |
| Age distribution of in-migrants | N/A | N/A | N/A |
| Exits per year by age | N/A | CfWI, 2013^[[15]](#footnote-15)^ | N/A |
| Total yearly enrolment in training programs | N/A | CfWI, 2013 | N/A |
| % of students completing training | N/A | CfWI, 2013 | N/A |
| % of graduates remaining in-jurisdiction | N/A | N/A | N/A |
| Head count of current supply | OECD Stat | OECD Stat | OECD Stat |
| Age distribution of current supply | N/A | N/A | OECD Stat |
| % of licensed workforce providing any patient care | OED Stat | OECD Stat | OECD Stat |
| Average hours worked/week by participating workforce | N/A | CfWI, 2013 | N/A |

1. Australian Bureau of Statistics (2013). *Australian Health Survey: Service Usage and Health Related Actions, 2011-12*. Canberra: Author. Retrieved from <http://www.abs.gov.au/ausstats/Subscriber.nsf/LookupAttach/4364.0.55.002Data+Cubes-26.03.133/$File/43640DO003_20112012.xls>. [↑](#footnote-ref-1)
2. Health Workforce Australia (2012) *Health Workforce 2025: Doctors, Nurses and Midwives Volume 1.* Canberra: Author. Available at <https://submissions.education.gov.au/forms/archive/2015_16_sol/documents/Attachments/Australian%20Nursing%20and%20Midwifery%20Accreditation%20Council%20%28ANMAC%29.pdf>. [↑](#footnote-ref-2)
3. Authors’ calculations based on analysis of public use microdata from the 2012 Canadian Community Health Survey (CCHS). See Statistics Canada (2016). *Canadian Community Health Survey: Annual Component*. Retrieved from <http://www23.statcan.gc.ca/imdb/p2SV.pl?Function=getSurvey&SDDS=3226&lang=en&db=imdb&adm=8&dis=2>. [↑](#footnote-ref-3)
4. See Canadian Institute for Health Information (2015). *National Physician Database, 2014-15 Annual Release*. Ottawa: CIHI. Retrieved from https://secure.cihi.ca/estore/productFamily.htm?locale=en&pf=PFC3268&lang=en [↑](#footnote-ref-4)
5. O’Brien-Pallas L, Tomblin Murphy G, Shamian J, Li X, Hayes L. (2012). Impact and determinants of nurse turnover: A pan-Canadian study. *Journal of Nursing Management, 18*(8): 1073-1086. [↑](#footnote-ref-5)
6. Tomblin Murphy G, Birch S, MacKenzie A, Alder R, Lethbridge L, Little L. (2012). Eliminating the shortage of Registered Nurses in Canada: An exercise in applied needs-based planning. *Health Policy, 105:192-202.*  [↑](#footnote-ref-6)
7. See Canadian Post-MD Education Registry (2015). *2014-2015 Annual Census of Post-MD Trainees*. Ottawa: CAPER. Retrieved from <http://caper.ca/~assets/documents/pdf_2014-15_CAPER_Census.pdf>. And Canadian Post-MD Education Registry (2013). *The National IMG Database Report*. Ottawa: CAPER. Retrieved from <http://caper.ca/~assets/documents/2012_CAPER_National_IMG_Database_Report.pdf>. [↑](#footnote-ref-7)
8. See CIHI (2015) *Regulated Nurses, 2014*. Ottawa: CIHI. Retrieved from <https://secure.cihi.ca/free_products/RegulatedNurses2014_Report_EN.pdf>. [↑](#footnote-ref-8)
9. Pong R (2011) *Putting away the stethoscope for good? Toward a new perspective on physician retirement.* Ottawa: CIHI. Retrieved from <https://secure.cihi.ca/free_products/HHR%20Physician%20Report_En_Web.pdf>. [↑](#footnote-ref-9)
10. Canadian Medical Association (2015). First Year Enrolment in Canadian Faculties of Medicine 1957/58 – 2014-15. Ottawa: CMA. Retrieved from <https://www.cma.ca/Assets/assets-library/document/en/advocacy/23-1st-yr-sex.pdf>. [↑](#footnote-ref-10)
11. Canadian Association of Midwives (2015). *Midwifery in Canada – Provinces/Territories*. Ottawa: CAM. Retrieved from <http://www.canadianmidwives.org/province/Alberta.html>. [↑](#footnote-ref-11)
12. National Physician Survey (2015). *2014 National Results by Age and Sex Group – Question 23*. Retrieved from <http://nationalphysiciansurvey.ca/wp-content/uploads/2014/08/2014-National-EN-Q23.pdf>. [↑](#footnote-ref-12)
13. Health Resources and Services Administration (2014) *The Future of the Nursing Workforce: National- and State-Level Projections, 2012-2025*. Washington: Department of Health and Human Services. Retrieved from <http://bhpr.hrsa.gov/healthworkforce/supplydemand/nursing/workforceprojections/nursingprojections.pdf>. [↑](#footnote-ref-13)
14. Health Resources and Services Administration (2013) *The US Nursing Workforce: Trends in Supply and Education*. Washington: Department of Health and Human Services. Retrieved From <http://bhpr.hrsa.gov/healthworkforce/supplydemand/nursing/nursingworkforce/nursingworkforcefullreport.pdf>. [↑](#footnote-ref-14)
15. Centre for Workforce Intelligence (2013) *Future nursing workforce projections: Starting the discussion*. London: CfWI. Retrieved from <http://www.cfwi.org.uk/publications/future-nursing-workforce-projections-starting%20the%20discussion>. [↑](#footnote-ref-15)
